# The sponge microbiome project

**DOI:** 10.1093/gigascience/gix077

**Published:** 2017-08-16

**Authors:** Lucas Moitinho-Silva, Shaun Nielsen, Amnon Amir, Antonio Gonzalez, Gail L Ackermann, Carlo Cerrano, Carmen Astudillo-Garcia, Cole Easson, Detmer Sipkema, Fang Liu, Georg Steinert, Giorgos Kotoulas, Grace P McCormack, Guofang Feng, James J Bell, Jan Vicente, Johannes R Björk, Jose M Montoya, Julie B Olson, Julie Reveillaud, Laura Steindler, Mari-Carmen Pineda, Maria V Marra, Micha Ilan, Michael W Taylor, Paraskevi Polymenakou, Patrick M Erwin, Peter J Schupp, Rachel L Simister, Rob Knight, Robert W Thacker, Rodrigo Costa, Russell T Hill, Susanna Lopez-Legentil, Thanos Dailianis, Timothy Ravasi, Ute Hentschel, Zhiyong Li, Nicole S Webster, Torsten Thomas

**Affiliations:** 1 Centre for Marine Bio-Innovation and School of Biological, Earth and Environmental Sciences, The University of New South Wales, Sydney, 2052, Australia; 2 Department of Pediatrics, University of California - San Diego, La Jolla, CA 92093, USA; 3 Department of Life and Environmental Sciences, Polytechnic University of Marche, Ancona, 60131, Italy; 4 School of Biological Sciences, University of Auckland, Auckland, New Zealand; 5 Halmos College of Natural Sciences and Oceanography, Nova Southeastern University, Dania Beach, FL 33004, USA; 6 Wageningen University, Laboratory of Microbiology, Stippeneng 4, 6708 WE Wageningen, The Netherlands; 7 State Key Laboratory of Microbial Metabolism and School of Life Sciences and Biotechnology, Shanghai Jiao Tong University, Shanghai 200240, P.R. China; 8 Hellenic Centre for Marine Research, Institute of Marine Biology, Biotechnology and Aquaculture, Thalassocosmos, 71500 Heraklion, Greece; 9 Zoology, School of Natural Sciences, Ryan Institute, National University of Ireland Galway, University Rd., Galway, Ireland; 10 School of Biological Sciences, Victoria University of Wellington, Wellington, New Zealand; 11 Hawaii Institute of Marine Biology, 46-007 Lilipuna Road, Kaneohe, HI 96744-1346; 12 Galvin Life Science Center, University of Notre Dame, Notre Dame, IN 46556, USA; 13 Ecological Networks and Global Change Group, Theoretical and Experimental Ecology Station, CNRS and Paul Sabatier University, Moulis, France; 14 Department of Biological Sciences, University of Alabama, Tuscaloosa, AL 35487, USA; 15 INRA, UMR1309 CMAEE; Cirad, UMR15 CMAEE, 34398 Montpellier, France; 16 Department of Marine Biology, Leon H. Charney School of Marine Sciences, University of Haifa, Haifa, Israel; 17 Australian Institute of Marine Science (AIMS), Townsville, 4810, Queensland, Australia; 18 Department of Zoology, George S. Wise Faculty of Life Sciences, Tel Aviv University, Tel Aviv 69978, Israel; 19 Department of Biology and Marine Biology, University of North Carolina Wilmington, Wilmington NC 28409, USA; 20 Institute for Chemistry and Biology of the Marine Environment (ICBM), Carl-von-Ossietzky and University Oldenburg, Schleusenstr. 1, 26382 Wilhelmshaven, Germany; 21 Department of Microbiology and Immunology, University of British Columbia, Canada, V6T 1Z3; 22 Department of Computer Science and Engineering, and Center for Microbiome Innovation, University of California - San Diego, La Jolla, CA 92093, USA; 23 Department of Ecology and Evolution, Stony Brook University, Stony Brook NY 11794, USA; 24 Institute for Bioengineering and Biosciences (IBB), Department of Bioengineering, IST, Universidade de Lisboa, Lisbon, Portugal; 25 Institute of Marine and Environmental Technology, University of Maryland Center for Environmental Science, 701 East Pratt Street, Baltimore, MD 21202, USA; 26 KAUST Environmental Epigenetic Program (KEEP), Division of Biological and Environmental Sciences & Engineering, King Abdullah University of Science and Technology, Thuwal, Kingdom of Saudi Arabia; 27 RD3 Marine Microbiology, GEOMAR Helmholtz Centre for Ocean Research, Kiel, and Christian-Albrechts-University of Kiel, Germany; 28 Australian Centre for Ecogenomics, School of Chemistry and Molecular Biosciences, University of Queensland, St Lucia, QLD, Australia

**Keywords:** marine sponges, archaea, bacteria, symbiosis, microbiome, 16S rRNA gene, microbial diversity

## Abstract

Marine sponges (phylum Porifera) are a diverse, phylogenetically deep-branching clade known for forming intimate partnerships with complex communities of microorganisms. To date, 16S rRNA gene sequencing studies have largely utilised different extraction and amplification methodologies to target the microbial communities of a limited number of sponge species, severely limiting comparative analyses of sponge microbial diversity and structure. Here, we provide an extensive and standardised dataset that will facilitate sponge microbiome comparisons across large spatial, temporal, and environmental scales. Samples from marine sponges (*n* = 3569 specimens), seawater (*n* = 370), marine sediments (*n* = 65) and other environments (*n* = 29) were collected from different locations across the globe. This dataset incorporates at least 268 different sponge species, including several yet unidentified taxa. The V4 region of the 16S rRNA gene was amplified and sequenced from extracted DNA using standardised procedures. Raw sequences (total of 1.1 billion sequences) were processed and clustered with (i) a standard protocol using QIIME closed-reference picking resulting in 39 543 operational taxonomic units (OTU) at 97% sequence identity, (ii) a *de novo* clustering using Mothur resulting in 518 246 OTUs, and (iii) a new high-resolution Deblur protocol resulting in 83 908 unique bacterial sequences. Abundance tables, representative sequences, taxonomic classifications, and metadata are provided. This dataset represents a comprehensive resource of sponge-associated microbial communities based on 16S rRNA gene sequences that can be used to address overarching hypotheses regarding host-associated prokaryotes, including host specificity, convergent evolution, environmental drivers of microbiome structure, and the sponge-associated rare biosphere.

## Data Description

### Purpose of data acquisition

Sponges (phylum Porifera) are an ancient metazoan clade [1], with more than 8500 formally described species [2]. Sponges are benthic organisms that have important ecological functions in aquatic habitats [3, 4]. Marine sponges are often found in symbiotic association with microorganisms, and these microbial communities can be very diverse and complex [5, 6]. Sponge symbionts perform a wide range of functional roles, including vitamin synthesis, production of bioactive compounds, and biochemical transformations of nutrients or waste products [7–9]. The diversity of microorganisms associated with sponges has been the subject of intense study (the search of “sponge microbial diversity” returned 348 publications in the Scopus database) [10]. Most of these studies were performed on individual species from restricted geographic regions [e.g., 11, 12]. A comparative assessment of these studies is often hindered by differences in sample processing and 16S rRNA gene sequencing. However, 2 recent studies incorporating a large number of sponge microbiomes (>30) [5, 13] revealed the potential of large-scale, standardised, high-throughput sequencing for gaining insights into the diversity and structure of sponge-associated microbial communities. The purpose of this global dataset is to provide a comprehensive 16S rRNA gene-based resource for investigating and comparing microbiomes more generally across the phylum Porifera.

### Sample collection, processing, and 16S rRNA gene sequencing

**Figure 1:**
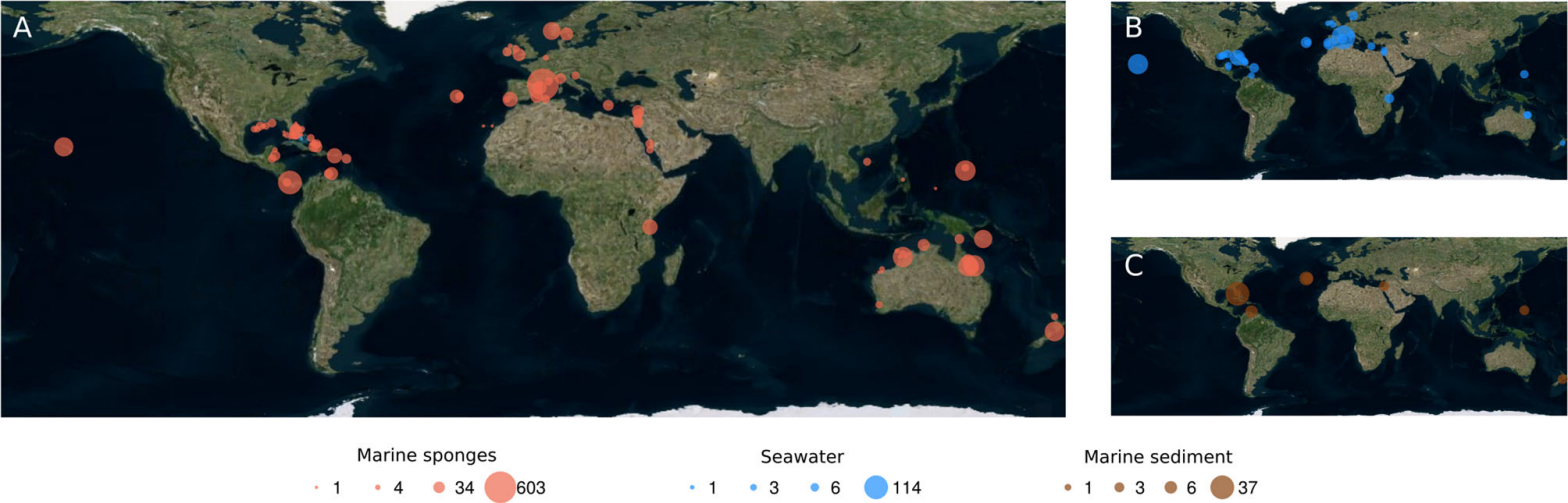
Global sample collection sites. Bubbles indicate collection sites of (**A**) marine sponges, (**B**) seawater, and (**C**) marine sediment samples. Bubble sizes are proportional to number of samples as indicated.

Sample collection and processing, species identification, and DNA extractions were conducted as previously described [13]. A total of 3569 sponge specimens were collected, representing at least 268 species, including several yet unidentified taxa (hereafter collectively referred to as species) (Supplementary Table S1). Of all species, 213 were represented by at least 3 specimens. *Carteriospongia foliascens* had the highest replication, comprising 150 individuals. Seawater (*n* = 370), sediment (*n* = 65), algae (*n* = 1), and echinoderm (*n* = 1) samples as well as biofilm swabs (*n* = 21) of rock surfaces were collected in close proximity to the sponges for comparative community analysis. Six negative control samples (sterile water) were processed to identify any potential contaminations. Of the samples included in this current dataset, 973 samples had been analysed previously [13]. Samples were collected from a wide range of geographical locations (Fig. 1; Supplementary Table S1). Total DNA was extracted as previously described [13] and used as templates to amplify and sequence the V4 region of the 16S rRNA gene using the standard procedures of the Earth Microbiome Project (EMP) [14 15].

### Processing of sequencing data

#### Clustering using the EMP standard protocols in QIIME

Raw sequences were demultiplexed and quality controlled following the recommendations of Bokulich et al. [16]. Quality-filtered, demultiplexed fastq files were processed using the default closed-reference pipeline from QIIME v. 1.9.1 (QIIME, RRID:SCR_008249). Briefly, sequences were matched against the GreenGenes reference database (v. 13_8 clustered at 97% similarity). Sequences that failed to align (e.g., chimeras) were discarded, which resulted in a final number of 300 140 110 sequences. Taxonomy assignments and the phylogenetic tree information were taken from the centroids of the reference sequence clusters contained in the GreenGenes reference database (Greengenes, RRID:SCR_002830). This closed-reference analysis allows for cross-dataset comparisons and direct comparison with the tens of thousands of other samples processed in the EMP and available via the Qiita database [17].

#### Clustering using Mothur

Quality-filtered, demultiplexed fastq files were also processed using Mothur v. 1.37.6 (Mothur, RRID:SCR_011947) [18] and Python v. 2.7 (Python Programming Language, RRID:SCR_008394) [19] custom scripts with modifications from previously established protocols [13]. Detailed descriptions and command outputs are available at the project notebook (see Availability of supporting data). Briefly, sequences were quality-trimmed to a maximum length of 100 bp. To minimize computational effort, the dataset was reduced to unique sequences, retaining total sequence counts. Sequences were aligned to the V4 region of the 16S rRNA gene sequences from the SILVA v. 123 database (SILVA, RRID:SCR_006423) [20]. Sequences that aligned at the expected positions were kept, and this dataset was again reduced to unique sequences. Further, singletons were removed from the dataset, and the remaining sequences were preclustered if they differed by 1 nucleotide position. Sequences classified as eukaryote, chloroplast, mitochondria, or unknown according to the Greengenes (v. 13_8 clustered at 99% similarity) [21] and SILVA taxonomies [22] were removed. Chimeras were identified with UCHIME (UCHIME, RRID:SCR_008057) [23] and removed. Finally, sequences were *de novo* clustered into operational taxonomic units (OTUs) using the furthest neighbour method at 97% similarity. Representative sequences of OTUs were retrieved based on the mean distance among the clustered sequences. Consensus taxonomies based on the SILVA, Greengenes, and RDP (v. 14_03 2015; Ribosomal Database Project, RRID:SCR_006633) [24] databases were obtained based on the classification of sequences clustered within each OTU. The inclusion of these taxonomies is helpful considering that they have substantial differences, as recently discussed [25]. For example, Greengenes and RDP have the taxon Poribacteria, a prominent sponge-enriched phylum [26], which did not exist in the SILVA version used.

#### De-noising using Deblur

Recently, sub-OTU methods that allow views of the data at single-nucleotide resolution have become available. One such method is Deblur [27], which is a de-noising algorithm for identification of the actual bacterial sequences present in a sample. Using an upper bound on the polymerase chain reaction and read-error rates, Deblur processes each sample independently and outputs the list of sequences and their frequencies in each sample, enabling single nucleotide resolution. For creating the deblurred biom table, quality-filtered, demultiplexed fasta files were used as input to Deblur using a trim length of 100 and min-reads of 25 (removing sOTUs with <25 reads total in all samples combined). Taxonomy was added to the resulting biom table using QIIME [28], RDP classifier [29], and Greengenes v. 13.8 [21].

#### Database metadata category enrichment

For enrichment analysis of metadata terms in a set of sequences, each unique metadata value is tested using both a binomial test and a ranksum test. All analysis is performed on a randomly subsampled (5000 reads/sample) table.

#### The binomial (presence/absence) *P*-value for enrichment calculated as follows

For a bacterial sequence s and metadata value v, denote *N* the total number of samples, O(s) the number of samples where s is present, K_v_(s) the number of samples with value v where s is present, and T(v) the total number of samples with value v.

\begin{eqnarray*}
P\hbox{-value} = {\it binomial}\_{\it cdf}
\left({\rm{T}} ({\rm v}) - {{\rm{K}}_{\rm{v}}} ({\rm s}), {\rm{T}} ({\rm v}), 1-P_{\rm{Null}} ({\rm s}) \right)
\end{eqnarray*}

where *P*_Null_(s) = O(s)/*N*

The ranksum (frequency aware) *P*-value is calculated using the Kruskal-Wallis test (implemented in scipy 0.19) as follows.

For a bacterial sequence s and metadata value v, denote by *F_v_*(*s*) the vector of relative frequencies of bacteria s in all samples with metadata value v, and denote by $\widehat {{F_v}( s )}$ the vector of relative frequencies of bacteria s in all samples with metadata other than v. The ranksum *P*-value is then calculated using the Kruskal-Wallis test for *F_v_*(*s*) and $\widehat {{F_v}( s )}$ and shown only if significantly enriched in samples containing v (i.e., rank difference of *F_v_*(*s*)—$\widehat {{F_v}( s )} $ > 0).

We have set up a webserver [30] that performs this enrichment analysis for user-defined sequence submissions. The code for the webserver is also available in Github for a local installation.

### Data description

**Figure 2:**
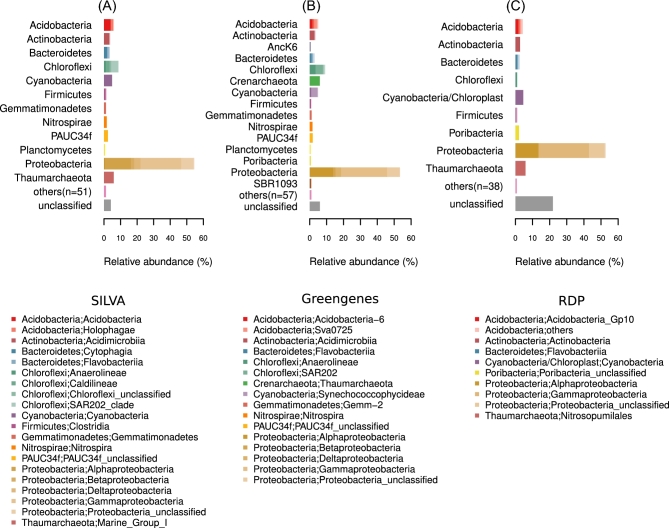
Microbial taxonomic profile of marine sponge samples processed with Mothur. (**A**) SILVA, (**B**) Greengenes, and (**C**) RDP taxonomies are shown. OTU sequence counts were grouped according to phylum and class. Taxa with relative abundances ≤0.5% were grouped as “others.” Classes with relative abundances >1% are shown in the legend (phylum “;” class). Relative abundances are represented on the x-axes.

The dataset covers 4033 samples with a total of 1 167 226 701 raw sequence reads. These sequence reads clustered into 39 543 OTUs using QIIME’s closed-reference processing, 518 246 OTUs from *de novo* clustering using Mothur (not filtered for OTU abundances), and 83 908 sOTUs using Deblur (with a filtering of at least 25 reads total per sOTU). We recommend that data users consider the differences in sequencing depths per sample and abundance filtering for certain downstream analyses, such as when calculating diversity estimates [16] and comparing OTU abundances across samples [31]. In terms of taxonomic diversity, most Mothur OTUs were assigned to the phylum Proteobacteria, although more than 60 different microbial phyla were recovered from the marine sponge samples according to SILVA (*n* = 63) and Greengenes classifications (*n* = 72) (Fig. 2).

### Potential uses

**Figure 3:**
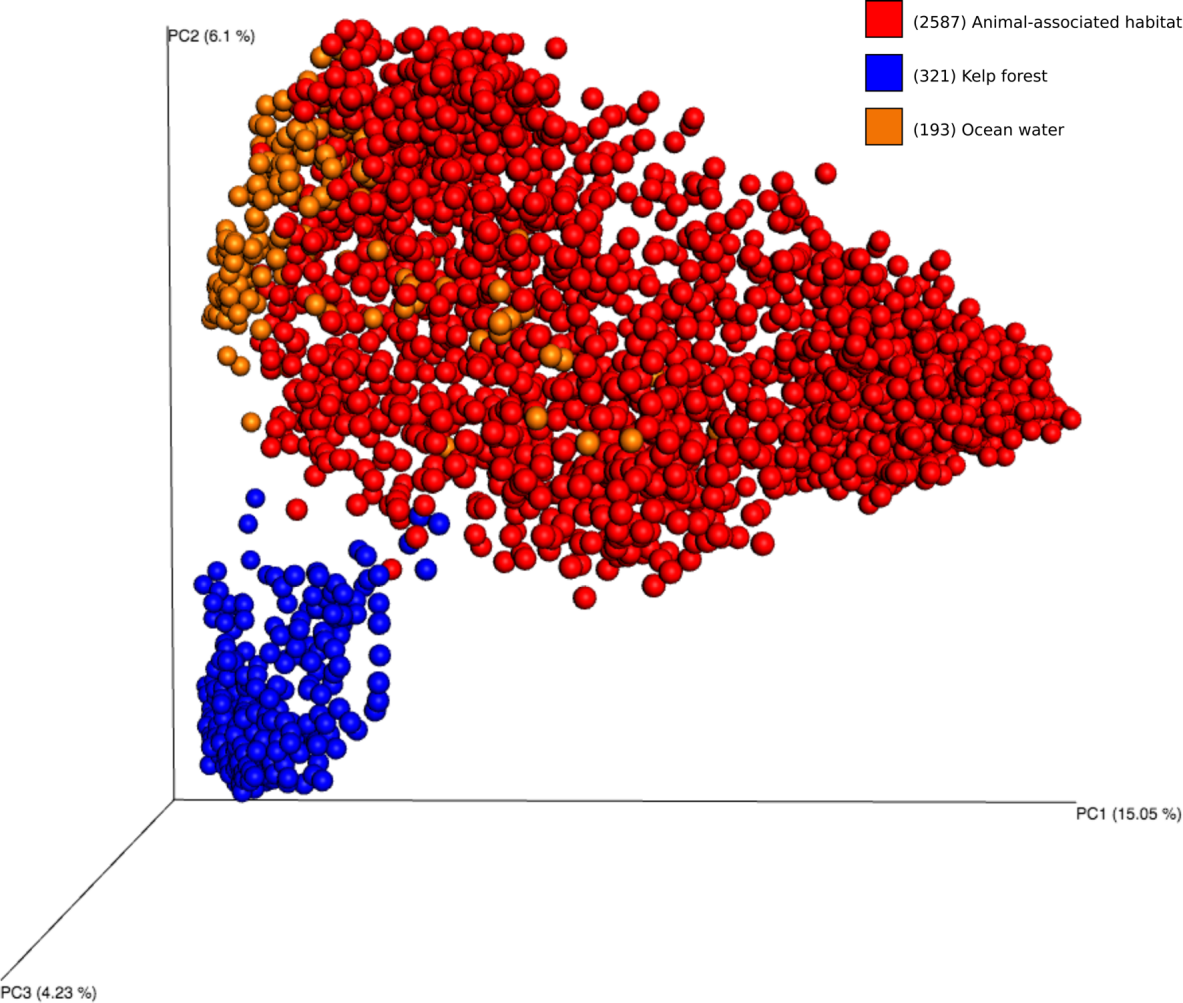
Unweighted UniFrac Principal Coordinates Analysis (PCA) of samples from sponges (“animal-associated habitat”), kelp forest, and ocean water. Samples were rarefying to 10 000 sequences per sample. A movie showing the PCA plot in 3D is provided in the supporting information.

This dataset can be utilised to assess a broad range of ecological questions pertaining to host-associated microbial communities generally or to sponge microbiology specifically. These include: (i) the degree of host specificity, (ii) the existence of biogeographic or environmental patterns, (iii) the relation of microbiomes to host phylogeny, (iv) the variability of microbiomes within or between host species, (v) symbiont co-occurrence patterns, and (vi) assessing the existence of a core sponge microbiome. An example of this type of analysis is shown in Fig. 3, where samples were clustered using unweighted UniFrac data [10] with a Principal Coordinates Analysis and visualization in Emperor [15] based on their origins from sponges, seawater, or kelps [17].

## Availability and requirements

Project name: The Sponge Microbiome Project

Project home page: www.spongeemp.com; https://github.com/amnona/SpongeEMP

Operating system(s): Unix

Programming language: Python and R

Other requirements: Python v. 2.7, Biopython v. 1.65, Python 3.5, R v. 3.2.2, Mothur v. 1.37.6, QIIME v. 1.9.1, Deblur

License: MIT

Any restrictions to use by non-academics: none

## Availability of supporting data

Raw sequence data were deposited in the European Nucleotide Archive (accession number: ERP020690). Quality-filtered, demultiplexed fastq files, QIIME resulting OTU tables are available at the Qiita database (Study ID: 10 793) [17]. The additional datasets that support the results of this article are available in the *GigaScience* repository, *Giga*DB [32] and include an OTU abundance matrix (the output “.shared” file from Mothur, which is tab delimited), an OTU taxonomic classification table (tab delimited text file), an OTU representative sequence FASTA file, a table of samples’ metadata, the biom files from QIIME and Deblur analyses, and the QIIME-generated tree file. The project workflow, Mothur commands, and additional scripts are available as HTML in *Giga*DB [32].

**Figure 4:**
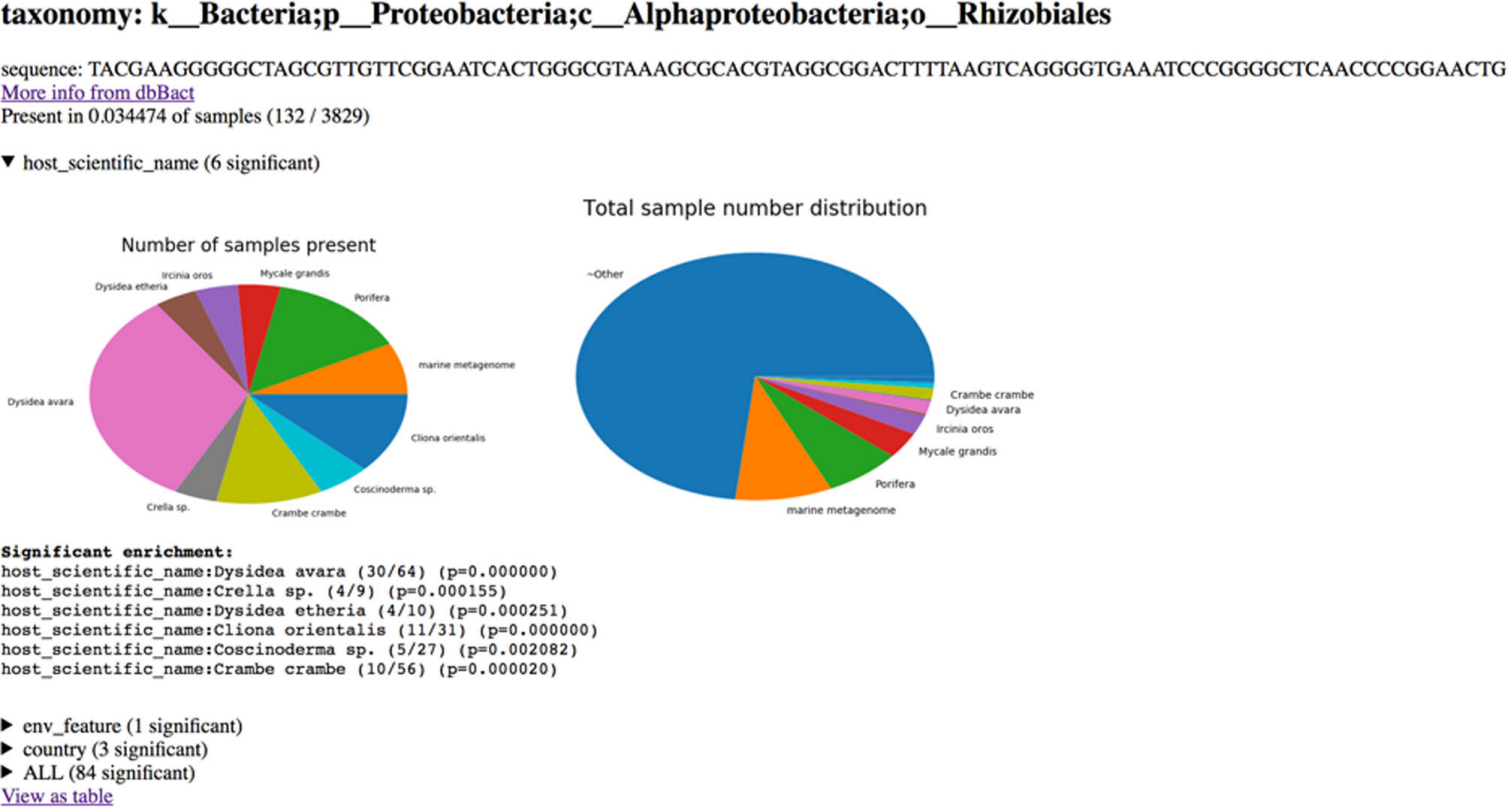
Output of the enrichment analysis through the online server www.spongeemp.com. Top line shows taxonomic assignment for the user-submitted sequence in the second line. Pie charts below show the total number of samples (right) and the number of samples where the submitted sequence is present (left) based on the scientific names of the host, followed by the significantly enriched host names containing the submitted sequence (using either presence/absence binomial test or relative frequency–based ranksum test). At the bottom, fields can be opened to show results of the enrichment analyses for other metadata types (e.g., country).

The deblurred dataset has also been uploaded to an online server [19] that supplies both html and REST-API access for querying bacterial sequences and obtaining the observed prevalence and enriched metadata categories where the sequence is observed (Figure 4). This allows an interactive view of which sequences are associated with which specific parameters, such as depth or salinity.

## Additional file

sample.metadata

## Abbreviations

EMP: Earth microbiome project; bp: base pairs; OTU: operational taxonomic unit; rRNA: ribosomal RNA.

## Funding

T.T. and N.S.W were funded by Australian Research Council Future Fellowships FT140100197 and FT120100480, respectively. T.T. received funds from the Gordon and Betty Moore Foundation. This work was also supported in part by the W.M. Keck Foundation and the John Templeton Foundation. R.K. received funding as a Howard Hughes Medical Institute Early Career Scientist.

## Competing interests

The authors declare that they have no competing interests.

## Author contributions

L.M.-S., N.S.W., and T.T. designed the study. C.A.G., D.S., F.L., G.S., G.K., G.McC., G.-F. F, J.J.B., J.V., J.R.B., J.M.M., J.R., L.S., M.C.P, M.V.M., M.W.T., N.S.W., P.P., P.M.E., P.J.S., R.L.S, R.W.T., R.C., R.T.H., S.L-L., T.D., T.R., U.H., and Z-Y. L. collected samples. C.A.G., D.S., J.V., J.R.B., L.S., M.C.P., M.W.T., N.S.W., P.M.E., R.L.S, R.W.T., S.L-L., and U.H. extracted DNA. G.L.A. and R.K. sequenced DNA. L.M.-S., S.N., A.A., A.G., G.L.A., and T.T. performed data processing and analysis. L.M.-S., N.S.W., and T.T. wrote the manuscript. All authors contributed to the writing of the manuscript.

## Supplementary Material

GIGA-D-17-00079_Original-Submission.pdf

GIGA-D-17-00079_Revision-1.pdf

Response-to-Reviewer-Comments_Original-Submission.pdf

Reviewer-1-Report-(Original-Submission).pdf

Reviewer-1-Report-(Revision-1).pdf

Reviewer-2-Report-(Original-Submission).pdf

Additional Files

## References

[1] Li CW, Chen JY, Hua TE Precambrian sponges with cellular structures. Science 1998;279(5352):879–82. 9452391 10.1126/science.279.5352.879

[2] Van Soest RWM, Boury-Esnault N, Vacelet J Global diversity of sponges (Porifera). PLoS One 2012;7(4):e35105. doi:10.1371/journal.pone.0035105. 22558119 PMC3338747

[3] Bell JJ The functional roles of marine sponges. Estuar Coast Shelf Sci 2008;79(3):341–53.

[4] De Goeij JM, Van Oevelen D, Vermeij MJA Surviving in a marine desert: the sponge loop retains resources within coral reefs. Science 2013;342(6154):108–10. 24092742 10.1126/science.1241981

[5] Schmitt S, Tsai P, Bell J Assessing the complex sponge microbiota: core, variable and species-specific bacterial communities in marine sponges. ISME J 2012;6(3):564–76. 21993395 10.1038/ismej.2011.116 PMC3280146

[6] Webster NS, Taylor MW, Behnam F Deep sequencing reveals exceptional diversity and modes of transmission for bacterial sponge symbionts. Environ Microbiol 2010;12(8):2070–82. 21966903 10.1111/j.1462-2920.2009.02065.x PMC2936111

[7] Siegl A, Kamke J, Hochmuth T Single-cell genomics reveals the lifestyle of *Poribacteria*, a candidate phylum symbiotically associated with marine sponges. ISME J 2011;5(1):61–70. 20613790 10.1038/ismej.2010.95 PMC3105677

[8] Taylor MW, Radax R, Steger D Sponge-associated microorganisms: evolution, ecology, and biotechnological potential. Microbiol Mol Biol Rev 2007;71(2):295–347. 17554047 10.1128/MMBR.00040-06 PMC1899876

[9] Wilson MC, Mori T, Ruckert C An environmental bacterial taxon with a large and distinct metabolic repertoire. Nature 2014;506(7486):58–62. 24476823 10.1038/nature12959

[10] Lozupone C, Knight R UniFrac: a new phylogenetic method for comparing microbial communities. Appl Environ Microbiol 2005;71(12):8228–35. 16332807 10.1128/AEM.71.12.8228-8235.2005 PMC1317376

[11] Moitinho-Silva L, Bayer K, Cannistraci CV Specificity and transcriptional activity of microbiota associated with low and high microbial abundance sponges from the Red Sea. Mol Ecol 2014;23(6):1348–63. 23957633 10.1111/mec.12365

[12] Montalvo NF, Hill RT Sponge-associated bacteria are strictly maintained in two closely related but geographically distant sponge hosts. Appl Environ Microbiol 2011;77(20):7207–16. 21856832 10.1128/AEM.05285-11 PMC3194858

[13] Thomas T, Moitinho-Silva L, Lurgi M Diversity, structure and convergent evolution of the global sponge microbiome. Nat Commun 2016;7:11870. doi:10.1038/ncomms11870. 27306690 PMC4912640

[14] Gilbert JA, Jansson JK, Knight R The Earth Microbiome project: successes and aspirations. BMC Biol 2014;12 1:69. doi:10.1186/s12915-014-0069-1. 25184604 PMC4141107

[15] Vazquez-Baeza Y, Pirrung M, Gonzalez A EMPeror: a tool for visualizing high-throughput microbial community data. Gigascience 2013;2(1):16. doi:10.1186/2047-217X-2-16. 24280061 PMC4076506

[16] Bokulich NA, Subramanian S, Faith JJ Quality-filtering vastly improves diversity estimates from Illumina amplicon sequencing. Nat Methods 2013;10(1):57–59. 23202435 10.1038/nmeth.2276 PMC3531572

[17] Marzinelli EM, Campbell AH, Zozaya Valdes E Continental-scale variation in seaweed host-associated bacterial communities is a function of host condition, not geography. Environ Microbiol 2015;17(10):4078–88. 26148974 10.1111/1462-2920.12972

[18] Schloss PD, Westcott SL, Ryabin T Introducing Mothur: open-source, platform-independent, community-supported software for describing and comparing microbial communities. Appl Environ Microbiol 2009;75(23):7537–41. 19801464 10.1128/AEM.01541-09 PMC2786419

[19] Sponge microbiome project deblurred dataset online server. http://www.spongeemp.com. Accessed 31 March 2017.

[20] Quast C, Pruesse E, Yilmaz P The SILVA ribosomal RNA gene database project: improved data processing and web-based tools. Nucleic Acids Res 2013;41(D1):D590–6. 23193283 10.1093/nar/gks1219 PMC3531112

[21] Desantis TZ, Hugenholtz P, Larsen N Greengenes, a chimera-checked 16S rRNA gene database and workbench compatible with ARB. Appl Environ Microbiol 2006;72(7):5069–72. 16820507 10.1128/AEM.03006-05 PMC1489311

[22] Yilmaz P, Parfrey LW, Yarza P The SILVA and All-species Living Tree Project (LTP)–taxonomic frameworks. Nucl Acids Res 2014;42(D1):D643–8. 24293649 10.1093/nar/gkt1209 PMC3965112

[23] Edgar RC, Haas BJ, Clemente JC UCHIME improves sensitivity and speed of chimera detection. Bioinformatics 2011;27(16):2194–200. 21700674 10.1093/bioinformatics/btr381 PMC3150044

[24] Cole JR, Wang Q, Fish JA Ribosomal database project: data and tools for high throughput rRNA analysis. Nucl Acids Res 2014;42(D1):D633–42. 24288368 10.1093/nar/gkt1244 PMC3965039

[25] Balvociute M, Huson DH SILVA, RDP, Greengenes, NCBI and OTT – how do these taxonomies compare? BMC Genomics 2017;18(S2):114. doi:10.1186/s12864-017-3501-4. 28361695 PMC5374703

[26] Fieseler L, Horn M, Wagner M Discovery of the novel candidate phylum "Poribacteria" in marine sponges. Appl Environ Microbiol 2004;70(6):3724–32. 15184179 10.1128/AEM.70.6.3724-3732.2004 PMC427773

[27] Amir A, Mcdonald D, Navas-Molina JA Deblur rapidly resolves single-nucleotide community sequence patterns. mSystems 2017;2(2). doi:10.1128/mSystems.00191-16. PMC5340863 28289731

[28] Caporaso JG, Kuczynski J, Stombaugh J QIIME allows analysis of high-throughput community sequencing data. Nat Methods 2010;7(5):335–6. 20383131 10.1038/nmeth.f.303 PMC3156573

[29] Wang Q, Garrity GM, Tiedje JM Naive Bayesian classifier for rapid assignment of rRNA sequences into the new bacterial taxonomy. Appl Environ Microbiol 2007;73(16):5261–7. 17586664 10.1128/AEM.00062-07 PMC1950982

[30] www.spongeemp.com.

[31] Mcmurdie PJ, Holmes S, Mchardy AC Waste not, want not: why rarefying microbiome data is inadmissible. PLoS Comput Biol 2014;10(4):e1003531. doi:10.1371/journal.pcbi.1003531.32. 24699258 PMC3974642

[32] Moitinho-Silva L, Nielsen S, Amir A Supporting data for “The sponge microbiome project.” GigaScience Database. 2017 10.5524/100332. PMC5632291 29020741

[33] SpongeEMP GitHub. https://github.com/amnona/SpongeEMP. Accessed 31 March 2017.

