# Erratum to: The sponge microbiome project

**DOI:** 10.1093/gigascience/giy145

**Published:** 2018-12-06

**Authors:** Lucas Moitinho-Silva, Shaun Nielsen, Amnon Amir, Antonio Gonzalez, Gail L Ackermann, Carlo Cerrano, Carmen Astudillo-Garcia, Cole Easson, Detmer Sipkema, Fang Liu, Georg Steinert, Giorgos Kotoulas, Grace P McCormack, Guofang Feng, James J Bell, Jan Vicente, Johannes R Björk, Jose M Montoya, Julie B Olson, Julie Reveillaud, Laura Steindler, Mari-Carmen Pineda, Maria V Marra, Micha Ilan, Michael W Taylor, Paraskevi Polymenakou, Patrick M Erwin, Peter J Schupp, Rachel L Simister, Rob Knight, Robert W Thacker, Rodrigo Costa, Russell T Hill, Susanna Lopez-Legentil, Thanos Dailianis, Timothy Ravasi, Ute Hentschel, Zhiyong Li, Nicole S Webster, Torsten Thomas

**Affiliations:** 1 Centre for Marine Bio-Innovation and School of Biological, Earth and Environmental Sciences, The University of New South Wales, Sydney, 2052, Australia; 2 Department of Pediatrics, University of California - San Diego, La Jolla, CA 92093, USA; 3 Department of Life and Environmental Sciences, Polytechnic University of Marche, Ancona, 60131, Italy; 4 School of Biological Sciences, University of Auckland, Auckland, New Zealand; 5 Halmos College of Natural Sciences and Oceanography, Nova Southeastern University, Dania Beach, FL 33004, USA; 6 Wageningen University, Laboratory of Microbiology, Stippeneng 4, 6708 WE Wageningen, The Netherlands; 7 State Key Laboratory of Microbial Metabolism and School of Life Sciences and Biotechnology, Shanghai Jiao Tong University, Shanghai 200240, P.R. China; 8 Hellenic Centre for Marine Research, Institute of Marine Biology, Biotechnology and Aquaculture, Thalassocosmos, 71500 Heraklion, Greece; 9 Zoology, School of Natural Sciences, Ryan Institute, National University of Ireland Galway, University Rd., Galway, Ireland; 10 School of Biological Sciences, Victoria University of Wellington, Wellington, New Zealand; 11 Hawaii Institute of Marine Biology, 46-007 Lilipuna Road, Kaneohe, HI 96744-1346; 12 Galvin Life Science Center, University of Notre Dame, Notre Dame, IN 46556, USA; 13 Ecological Networks and Global Change Group, Theoretical and Experimental Ecology Station, CNRS and Paul Sabatier University, Moulis, France; 14 Department of Biological Sciences, University of Alabama, Tuscaloosa, AL 35487, USA; 15 INRA, UMR1309 CMAEE; Cirad, UMR15 CMAEE, 34398 Montpellier, France; 16 Department of Marine Biology, Leon H. Charney School of Marine Sciences, University of Haifa, Haifa, Israel; 17 Australian Institute of Marine Science (AIMS), Townsville, 4810, Queensland, Australia; 18 Department of Zoology, George S. Wise Faculty of Life Sciences, Tel Aviv University, Tel Aviv 69978, Israel; 19 Department of Biology and Marine Biology, University of North Carolina Wilmington, Wilmington NC 28409, USA; 20 Institute for Chemistry and Biology of the Marine Environment (ICBM), Carl-von-Ossietzky and University Oldenburg, Schleusenstr. 1, 26382 Wilhelmshaven, Germany; 21 Department of Microbiology and Immunology, University of British Columbia, Canada, V6T 1Z3; 22 Department of Computer Science and Engineering, and Center for Microbiome Innovation, University of California - San Diego, La Jolla, CA 92093, USA; 23 Department of Ecology and Evolution, Stony Brook University, Stony Brook NY 11794, USA; 24 Institute for Bioengineering and Biosciences (IBB), Department of Bioengineering, IST, Universidade de Lisboa, Lisbon, Portugal; 25 Institute of Marine and Environmental Technology, University of Maryland Center for Environmental Science, 701 East Pratt Street, Baltimore, MD 21202, USA; 26 KAUST Environmental Epigenetic Program (KEEP), Division of Biological and Environmental Sciences & Engineering, King Abdullah University of Science and Technology, Thuwal, Kingdom of Saudi Arabia; 27 RD3 Marine Microbiology, GEOMAR Helmholtz Centre for Ocean Research, Kiel, and Christian-Albrechts-University of Kiel, Germany; 28 Australian Centre for Ecogenomics, School of Chemistry and Molecular Biosciences, University of Queensland, St Lucia, QLD, Australia

“The sponge microbiome project” Lucas Moitinho-Silva et al. GigaScience, 6, 2017; doi: 10.1093/gigascience/gix077.

A formula was incorrect upon initial publication. The formula “p-value = binomial_cdf (T(v)-Kv(s), T(v), PNull (s))” should instead be “p-value = binomial_cdf (T(v)-Kv(s), T(v), 1-PNull (s)).”

This has now been corrected.

