# Supplementary material for: The sponge microbiome project
Source: Gigascience. 2017 Aug 16;6(10):gix077. doi: 10.1093/gigascience/gix077 (PMC5632291; doi:10.1093/gigascience/gix077)
Supplement: GIGA-D-17-00079_Original-Submission.pdf [file gix077_giga-d-17-00079_original-submission.pdf]

Manuscript Click here to download Manuscript SMP.gigascience.final.docx

# The sponge microbiome project

Lucas Moitinho-Silva[1], Shaun Nielsen[1], Amnon Amir[2], Antonio Gonzalez[2], Gail L. Ackermann[2], Carlo Cerrano[3], Carmen Astudillo-Garcia[4], Cole Easson[5], Detmer Sipkema[6], Fang Liu[7], Georg Steinert[6], Giorgos Kotoulas[7], Grace P. McCormack[8], Guofang Feng[9], James J. Bell[10], Jan Vicente[11], Johannes R Björk[12], Jose M. Montoya[13], Julie B. Olson[14], Julie Reveillaud[15], Laura Steindler[16], Mari-Carmen Pineda[17], Maria V. Marra[9], Micha Ilan[18], Michael W. Taylor[3], Paraskevi Polymenakou[8], Patrick M. Erwin[19], Peter J. Schupp[20], Rachel L. Simister[21], Rob Knight[2,22], Robert W. Thacker[23], Rodrigo Costa[24], Russell T. Hill[25], Susanna Lopez-Legentil[19], Thanos Dailianis[8], Timothy Ravasi[26], Ute Hentschel[27], Zhiyong Li[6], Nicole S. Webster[17, 28] and Torsten Thomas[1,*]

[1]Centre for Marine Bio-Innovation and School of Biological, Earth and Environmental Sciences, The University of New South Wales, Sydney, 2052, Australia

[2]Department of Pediatrics, University of California - San Diego, La Jolla, CA 92093, USA

[3]Department of Life and Environmental Sciences, Polytechnic University of Marche, Ancona, 60131, Italy

[4]School of Biological Sciences, University of Auckland, Auckland, New Zealand

[5]Halmos College of Natural Sciences and Oceanography, Nova Southeastern University, Dania Beach, FL 33004, USA

[6]Wageningen University, Laboratory of Microbiology, Stippeneng 4, 6708 WE Wageningen, The Netherlands

[7]Hellenic Centre for Marine Research, Institute of Marine Biology, Biotechnology and Aquaculture, Thalassocosmos 71500 Heraklion Greece

[8]Zoology, School of Natural Sciences, Ryan Institute, National University of Ireland Galway, University Rd., Galway, Ireland

[9]State Key Laboratory of Microbial Metabolism and School of Life Sciences and Biotechnology, Shanghai Jiao Tong University, Shanghai 200240, P.R. China

[10]School of Biological Sciences, Victoria University of Wellington, Wellington, New Zealand

[11]Hawaii Institute of Marine Biology, 46-007 Lilipuna Road, Kaneohe, HI 96744-1346

[12]Galvin Life Science Center, University of Notre Dame, Notre Dame, IN 46556, USA and Ecological Networks and Global Change Group, Theoretical and Experimental Ecology Station, CNRS, Moulis, France

[13]Ecological Networks and Global Change Group, Theoretical and Experimental Ecology Station, CNRS and Paul Sabatier University, Moulis, France

[14]Department of Biological Sciences, University of Alabama, Tuscaloosa, AL 35487, USA

[15]INRA, UMR1309 CMAEE; Cirad, UMR15 CMAEE, 34398 Montpellier, France

[16]Department of Marine Biology, Leon H. Charney School of Marine Sciences, University of Haifa, Haifa, Israel

[17]Australian Institute of Marine Science (AIMS), Townsville, 4810, Queensland, Australia

[18]Department of Zoology, George S. Wise Faculty of Life Sciences, Tel Aviv University, Tel Aviv 69978, Israel

[19]Department of Biology and Marine Biology, University of North Carolina Wilmington, Wilmington NC 28409, USA

[20]Institute for Chemistry and Biology of the Marine Environment (ICBM), Carl-von-Ossietzky and University Oldenburg, Schleusenstr. 1, 26382 Wilhelmshaven, Germany

[21]Department of microbiology and immunology, University of British Columbia, Canada, V6T 1Z3

[22]Department of Computer Science and Engineering, and Center for Microbiome Innovation, University of California - San Diego, La Jolla, CA 92093, USA

[23]Department of Ecology and Evolution, Stony Brook University, Stony Brook NY 11794, USA

[24]Institute for Bioengineering and Biosciences (IBB), Department of Bioengineering, IST, Universidade de Lisboa, Lisbon, Portugal

[25]Institute of Marine and Environmental Technology, University of Maryland Center for Environmental Science, 701 East Pratt Stree, Baltimore, MD 21202, USA

[26]KAUST Environmental Epigenetic Program (KEEP), Division of Biological and Environmental Sciences & Engineering, King Abdullah University of Science and Technology, Thuwal, Kingdom of Saudi Arabia

[27]RD3 Marine Microbiology, GEOMAR Helmholtz Centre for Ocean Research, Kiel, and Christian-Albrechts-University of Kiel, Germany

[28]Australian Centre for Ecogenomics, School of Chemistry and Molecular Biosciences, University of Queensland, St Lucia, QLD, Australia

*Corresponding author: t.thomas@unsw.edu.au; Centre for Marine Bio-Innovation, UNSW, Sydney, Australia

## Abstract

*Background:* Marine sponges (phylum Porifera) are a diverse, phylogenetically deep-branching clade known for forming intimate partnerships with complex communities of microorganisms. To date, 16S rRNA gene sequencing studies have largely utilised different extraction and amplification methodologies to target the microbial communities of a limited number of sponge species, severely limiting comparative analyses of sponge microbial diversity and structure. Here, we provide an extensive and standardised dataset that will facilitate sponge microbiome comparisons across large spatial, temporal and environmental scales.

*Findings:* Samples from marine sponges (n=3568 specimens), seawater (n=370), marine sediments (n=65) and other environments (n=29) were collected from different locations across the globe. This dataset incorporates at least 269 different sponge species, including several yet unidentified taxa. The V4 region of the 16S rRNA gene was amplified and sequenced from extracted DNA using standardised procedures. Raw sequences (total of 1.1 billion sequences) were processed and clustered with a) a standard protocol using QIIME closed-reference picking resulting in 39,543 Operational Taxonomic Units (OTU) at 97% sequence identity, b) a *de novo* protocol using Mothur resulting in 518,246 OTUs, and c) a new high-resolution Deblur protocol resulting in 83,908 unique bacterial sequences. Abundance tables, representative sequences, taxonomic classifications and metadata are provided.

*Conclusions:* This dataset represents a comprehensive resource of sponge-associated microbial communities based on 16S rRNA gene sequences that can be used to address overarching hypotheses regarding host-associated prokaryotes, including host-specificity, convergent evolution, environmental drivers of microbiome structure and the sponge-associated rare biosphere.

Keywords: Marine sponges, Archaea, Bacteria, Symbiosis, Microbiome, 16S rRNA gene, Microbial diversity

## Data Description

### *Purpose of data acquisition*

Sponges (phylum Porifera) are an ancient metazoan clade [1], with more than 8,500 formally described species [2]. Sponges are benthic organisms that have important ecological functions in aquatic habitats [3, 4]. Marine sponges are often found in symbiotic association with microorganisms and these microbial communities can be very diverse and complex [5, 6]. Sponge symbionts perform a wide range of functional roles, including vitamin synthesis, production of bioactive compounds and biochemical transformations of nutrients or waste products [7-9]. The diversity of microorganisms associated with sponges has been the subject of intense study (the search of "sponge microbial diversity" returned 348 publications in Scopus database [10]. Most of these studies were performed on individual species from restricted geographic regions [e.g., 11, 12]. A comparative assessment of these studies is often hindered by differences in sample processing and 16S rRNA gene sequencing. However, two recent studies incorporating a large number of sponge microbiomes (> 30) [5, 13] revealed the potential of large-scale, standardised, high-throughput sequencing for gaining unique insights into the diversity and structure of sponge-associated microbial communities. The purpose of this global dataset is to provide a comprehensive 16S rRNA gene-based resource for investigating and comparing microbiomes more generally across the phylum Porifera.

### *Sample collection, processing and 16S rRNA gene sequencing*

Sample collection and processing, species identification and DNA extractions were conducted as previously described [13]. A total of 3568 sponge specimens were collected, representing at least 268 species, including several yet unidentified taxa (hereafter collectively referred to as species) (Supplementary Table S1). Of the total species, 213 were represented by at least three specimens. *Carteriospongia foliascens* had the highest replication comprising 150 individuals. Seawater (n=370), sediment (n=65), algae (n=1) and echinoderm (n=1) samples as well as biofilm swabs (n=21) of rock surfaces were collected in close proximity to the sponges for comparative community analysis. Six negative control samples (sterile water) were processed to identify any potential contaminations. Of the samples included in this current dataset, 973 samples had been analysed previously [13]. Samples were collected from a wide range of geographical locations (Figure 1 and Supplementary Table S1). Total DNA was extracted as previously described [13] and used as templates to amplify and sequence the V4 region of the 16S rRNA gene using the standard procedures of the EMP [14, 15].

*Processing of sequencing data*

Clustering using the EMP standard protocols in QIIME:

Quality-filtered, demultiplexed fastq files were processed using the default closed-reference pipeline from QIIME v. 1.9.1, providing the EMP standard method for cross-dataset comparisons and allowing direct comparison with the tens of thousands of other samples processed in the EMP and available via the Qiita database [16]. Briefly, sequences were matched against GreenGenes (v. 13_8 at 97% similarity) reference database. Sequences that failed to align were discarded. Taxonomy for each sequence was taken from the cluster to which it aligned.

Clustering using Mothur:

Quality-filtered, demultiplexed fastq files were also processed using mothur v. 1.37.6 [17] and Python v. 2.7 [18] custom scripts with modifications from previously established protocols [13]. Detailed descriptions and command outputs are available at the project notebook (see Availability of supporting data). Briefly, sequences were quality-trimmed to a maximum length of 100 bp. To minimize computational effort, the dataset was reduced to unique sequences, retaining total sequence counts. Sequences were aligned to the V4 region of the 16S rRNA gene sequences from the SILVA v 123 database [19]. Sequences that aligned at the expected positions were kept and this dataset was again reduced to unique sequences. Further, singletons were removed from the dataset and remaining sequences were pre-clustered if they differed by one nucleotide position. Sequences classified as eukaryote, chloroplast, mitochondria or unknown according to the Greengenes (v. 13_8 at 99% similarity) [20] and SILVA taxonomies [21] were removed. Chimeras were identified with UCHIME [22] and removed. Finally, sequences were *de novo* clustered into Operational Taxonomic Units (OTUs) using the furthest neighbour method at 97% similarity. Representative sequences of OTUs were retrieved based on the mean distance among the clustered sequences. Consensus taxonomies based on the SILVA, Greengenes and RDP (v. 14_032015) [23] databases were obtained based on the classification of sequences clustered within each OTU.

De-noising using Deblur:

Recently, sub-OTU methods that allow views of the data at single-nucleotide resolution have become available. One such methods is Deblur [24], which is a denoising algorithm for identification of actual bacterial sequences present in a sample. Using an upper bound on the PCR and read-error

rates, Deblur processes each sample independently and outputs the list of sequences and their frequencies in each sample, enabling single nucleotide resolution. For creating the deblurred biom table, quality filtered, demultiplexed fasta files were used as input to Deblur using a trim length of 100, and min-reads of 25 (removing sOTUs with < 25 reads total in all samples combined). Taxonomy was added to resulting biom table using QIIME [25], RDP classifier [26] and Greengenes 13.8 [20].

Database metadata category enrichment:

For enrichment analysis of metadata terms in a set of sequences, each unique metadata value is tested using a binomial test. For a bacterial sequence s and metadata value v, denote N the total number of samples, O(s) the number of samples where s is present, $K_v(s)$ the number of sample with value v where s is present, and T(v) the total number of samples with value v. The p-value for enrichment was then calculated as:

$$\text{p-value} = \textit{binomial\_cdf}\,(\,T(v)-K_v(s),\ T(v),\ P_{Null}(s)\,)$$

where $P_{Null}(s) = O(s) / N$

We have set up a webserver (www.spongeemp.com) that performs this enrichment analysis for user-defined sequence submissions. The code for the webserver is also available in Github [27] for a local installation.

*Data description*

The dataset covers 4032 samples with a total of 1,167,226,701 raw sequence reads. These sequence reads clustered into 39,543 OTUs using QIIME's closed-reference processing, 518,246 OTUs from *de novo* clustering using Mothur (not filtered for OTU abundances), and 83,908 sOTUs using Deblur (with a filtering of at least 25 reads total per sOTU). We recommend that data users consider the differences in sequencing depths per sample and abundance filtering for certain downstream analyses, such as when calculating diversity estimates [28] and comparing OTU abundances across samples [29]. In terms of taxonomic diversity, most Mothur OTUs were assigned to the phylum Proteobacteria, although more than 60 different microbial phyla were recovered from the marine sponge samples according to SILVA (n=63) and Greengenes classifications (n=72) (Figure 2).

## Potential uses

This dataset can be utilised to assess a broad range of ecological questions pertaining to host-associated microbial communities generally or to sponge microbiology specifically. These include: i) the degree of host-specificity, ii) the existence of biogeographic or environmental patterns, iii) the relation of microbiomes to host phylogeny, iv) the variability of microbiomes within or between host species, v) symbiont co-occurrence patterns as well as vi) assessing the existence of a core sponge microbiome. An example of this type of analysis is shown in Figure 3, where samples were clustered using unweighted UniFrac data [30] with a Principal Coordinate Analysis and visualization in Emperor [31] based on their origins from sponges, seawater or kelps [32].

## Availability and requirements

Project name: The Sponge Microbiome Project

Project home page: GigaScience repository; www.spongeemp.com; https://github.com/amnona/SpongeEMP

Operating system(s): Unix

Programming language: Python and R

Other requirements: Python v. 2.7, Biopython v. 1.65, Python 3.5, R v. 3.2.2, mothur v. 1.37.6, QIIME v. 1.9.1, Deblur

License: MIT

Any restrictions to use by non-academics: None

## Availability of supporting data

Raw sequence data were deposited in the European Nucleotide Archive (accession numbers: ERP020690). Quality-filtered, demultiplexed fastq files, Deblur and QIIME resulting OTU tables are available at Qiita database [16] (Study ID: 10793). The additional datasets that support the results of

this article are available in the GigaScience repository **(DOI of the dataset)** and include an OTU abundance matrix (the output ".shared" file from mothur, which is tab delimited), an OTU taxonomic classification table (tab delimited text file), an OTU representative sequence FASTA file, and a table of samples' metadata. The project workflow, mothur commands and additional scripts are available as HTML TiddlyWiki notebook [33], which is viewed in any browser **(DOI of the workflow)**.

The deblurred dataset has also been uploaded to an online server [34] that supplies both html and REST-API access for querying bacterial sequences and obtaining the observed prevalence and enriched metadata categories where the sequence is observed (Figure 4). This allows an interactive view of which sequences are associated with which specific parameters, such as depth or salinity.

## List of abbreviations

bp: base pairs

OTU: operational taxonomic unit

rRNA: ribosomal RNA

## Competing interests

The authors declare that they have no competing interests.

## Funding

T.T. and N.S.W were funded by an Australian Research Council Future Fellowship FT140100197 and FT120100480, respectively. T.T. received funds from the Gordon and Betty Moore Foundation. This work was also supported in part by the W.M. Keck Foundation and the John Templeton Foundation. R.K. received funding as a Howard Hughes Medical Institute Early Career Scientist.

## Authors' contributions

L.M.-S., N.S.W. and T.T. designed the study. C.A.G., D.S., F.L., G.S., G.K., G.McC., G.-F. F, J.J.B., J.V., J.R.B., J.M.M., J.R., L.S., M.C.P, M.V.M., M.W.T., N.S.W., P.P., P.M.E., P.J.S., R.L.S, R.W.T., R.C., R.T.H., S.L-L., T.D., T.R., U.H. and Z-Y. L. collected samples. C.A.G., D.S., J.V., J.R.B., L.S., M.C.P., M.W.T., N.S.W., P.M.E., R.L.S, R.W.T., S.L-L. and U.H. extracted DNA. G.L.A. and R.K. sequenced DNA. L.M.-S., S.N., A.A., A.G., G.L.A. and T.T. performed data processing and analysis. L.M.-S., N.S.W. and T.T. wrote the manuscript. All authors contributed to the writing of the manuscript.

## References

1. Li CW, Chen JY and Hua TE. Precambrian sponges with cellular structures. Science. 1998;279 5352:879-82.
2. Van Soest RW, Boury-Esnault N, Vacelet J, Dohrmann M, Erpenbeck D, De Voogd NJ, et al. Global diversity of sponges (Porifera). PLoS One. 2012;7 4:e35105. doi:10.1371/journal.pone.0035105.
3. Bell JJ. The functional roles of marine sponges. Estuar Coast Shelf S. 2008;79 3:341-53.
4. de Goeij JM, van Oevelen D, Vermeij MJ, Osinga R, Middelburg JJ, de Goeij AF, et al. Surviving in a marine desert: the sponge loop retains resources within coral reefs. Science. 2013;342 6154:108-10. doi:10.1126/science.1241981.
5. Schmitt S, Tsai P, Bell J, Fromont J, Ilan M, Lindquist N, et al. Assessing the complex sponge microbiota: core, variable and species-specific bacterial communities in marine sponges. ISME J. 2012;6 3:564-76. doi:10.1038/ismej.2011.116.
6. Webster NS, Taylor MW, Behnam F, Lucker S, Rattei T, Whalan S, et al. Deep sequencing reveals exceptional diversity and modes of transmission for bacterial sponge symbionts. Environ Microbiol. 2010;12 8:2070-82. doi:10.1111/j.1462-2920.2009.02065.x.
7. Siegl A, Kamke J, Hochmuth T, Piel J, Richter M, Liang C, et al. Single-cell genomics reveals the lifestyle of Poribacteria, a candidate phylum symbiotically associated with marine sponges. ISME J. 2011;5 1:61-70. doi:10.1038/ismej.2010.95.
8. Taylor MW, Radax R, Steger D and Wagner M. Sponge-associated microorganisms: evolution, ecology, and biotechnological potential. Microbiol Mol Biol Rev. 2007;71 2:295-347. doi:10.1128/MMBR.00040-06.
9. Wilson MC, Mori T, Ruckert C, Uria AR, Helf MJ, Takada K, et al. An environmental bacterial taxon with a large and distinct metabolic repertoire. Nature. 2014;506 7486:58-62. doi:10.1038/nature12959.
10. Scopus database. https://www.scopus.com/. Accessed 1 Dec 2016.
11. Moitinho-Silva L, Bayer K, Cannistraci CV, Giles EC, Ryu T, Seridi L, et al. Specificity and transcriptional activity of microbiota associated with low and high microbial abundance sponges from the Red Sea. Mol Ecol. 2014;23 6:1348-63. doi:10.1111/mec.12365.
12. Montalvo NF and Hill RT. Sponge-associated bacteria are strictly maintained in two closely related but geographically distant sponge hosts. Appl Environ Microbiol. 2011;77 20:7207-16. doi:10.1128/AEM.05285-11.

13. Thomas T, Moitinho-Silva L, Lurgi M, Bjork JR, Easson C, Astudillo-Garcia C, et al. Diversity, structure and convergent evolution of the global sponge microbiome. Nat Commun. 2016;7:11870. doi:10.1038/ncomms11870.
14. Gilbert JA, Jansson JK and Knight R. The Earth Microbiome project: successes and aspirations. BMC Biol. 2014;12:69. doi:10.1186/s12915-014-0069-1.
15. EMP 16S Illumina amplicon protocol. http://www.earthmicrobiome.org/emp-standard-protocols/16s/. Accessed 1 Dec 2016.
16. Qiita database http://qiita.microbio.me/. Accessed 31 Mar 2017.
17. Schloss PD, Westcott SL, Ryabin T, Hall JR, Hartmann M, Hollister EB, et al. Introducing mothur: open-source, platform-independent, community-supported software for describing and comparing microbial communities. Appl Environ Microbiol. 2009;75 23:7537-41. doi:10.1128/AEM.01541-09.
18. Python. https://www.python.org/. Accessed 31 Mar 2017.
19. Quast C, Pruesse E, Yilmaz P, Gerken J, Schweer T, Yarza P, et al. The SILVA ribosomal RNA gene database project: improved data processing and web-based tools. Nucleic Acids Res. 2013;41 Database issue:D590-6. doi:10.1093/nar/gks1219.
20. DeSantis TZ, Hugenholtz P, Larsen N, Rojas M, Brodie EL, Keller K, et al. Greengenes, a chimera-checked 16S rRNA gene database and workbench compatible with ARB. Appl Environ Microbiol. 2006;72 7:5069-72. doi:10.1128/AEM.03006-05.
21. Yilmaz P, Parfrey LW, Yarza P, Gerken J, Pruesse E, Quast C, et al. The SILVA and "All-species Living Tree Project (LTP)" taxonomic frameworks. Nucleic Acids Res. 2014;42 Database issue:D643-8. doi:10.1093/nar/gkt1209.
22. Edgar RC, Haas BJ, Clemente JC, Quince C and Knight R. UCHIME improves sensitivity and speed of chimera detection. Bioinformatics. 2011;27 16:2194-200. doi:10.1093/bioinformatics/btr381.
23. Cole JR, Wang Q, Fish JA, Chai B, McGarrell DM, Sun Y, et al. Ribosomal Database Project: data and tools for high throughput rRNA analysis. Nucleic Acids Res. 2014;42 Database issue:D633-42. doi:10.1093/nar/gkt1244.
24. Amir A, McDonald D, Navas-Molina JA, Kopylova E, Morton JT, Zech Xu Z, et al. Deblur Rapidly Resolves Single-Nucleotide Community Sequence Patterns. mSystems. 2017;2 2 doi:10.1128/mSystems.00191-16.
25. Caporaso JG, Kuczynski J, Stombaugh J, Bittinger K, Bushman FD, Costello EK, et al. QIIME allows analysis of high-throughput community sequencing data. Nat Methods. 2010;7 5:335-6. doi:10.1038/nmeth.f.303.
26. Wang Q, Garrity GM, Tiedje JM and Cole JR. Naive Bayesian classifier for rapid assignment of rRNA sequences into the new bacterial taxonomy. Appl Environ Microbiol. 2007;73 16:5261-7. doi:10.1128/AEM.00062-07.
27. SpongeEMP GitHub. https://github.com/amnona/SpongeEMP. Accessed 31 Mar 2017.
28. Bokulich NA, Subramanian S, Faith JJ, Gevers D, Gordon JI, Knight R, et al. Quality-filtering vastly improves diversity estimates from Illumina amplicon sequencing. Nat Methods. 2013;10 1:57-9. doi:10.1038/nmeth.2276.
29. McMurdie PJ and Holmes S. Waste not, want not: why rarefying microbiome data is inadmissible. PLoS Comput Biol. 2014;10 4:e1003531. doi:10.1371/journal.pcbi.1003531.
30. Lozupone C and Knight R. UniFrac: a new phylogenetic method for comparing microbial communities. Appl Environ Microbiol. 2005;71 12:8228-35. doi:10.1128/AEM.71.12.8228-8235.2005.
31. Vazquez-Baeza Y, Pirrung M, Gonzalez A and Knight R. EMPeror: a tool for visualizing high-throughput microbial community data. Gigascience. 2013;2 1:16. doi:10.1186/2047-217X-2-16.
32. Marzinelli EM, Campbell AH, Zozaya Valdes E, Verges A, Nielsen S, Wernberg T, et al. Continental-scale variation in seaweed host-associated bacterial communities is a function of

host condition, not geography. Environ Microbiol. 2015;17 10:4078-88. doi:10.1111/1462-2920.12972.

33. TiddlyWiki. http://tiddlywiki.com/. Accessed 31 Mar 2017.

34. Sponge microbiome project deblurred dataset online server. www.spongeemp.com. Accessed 31 Mar 2017.

## Figures

Figure 1.

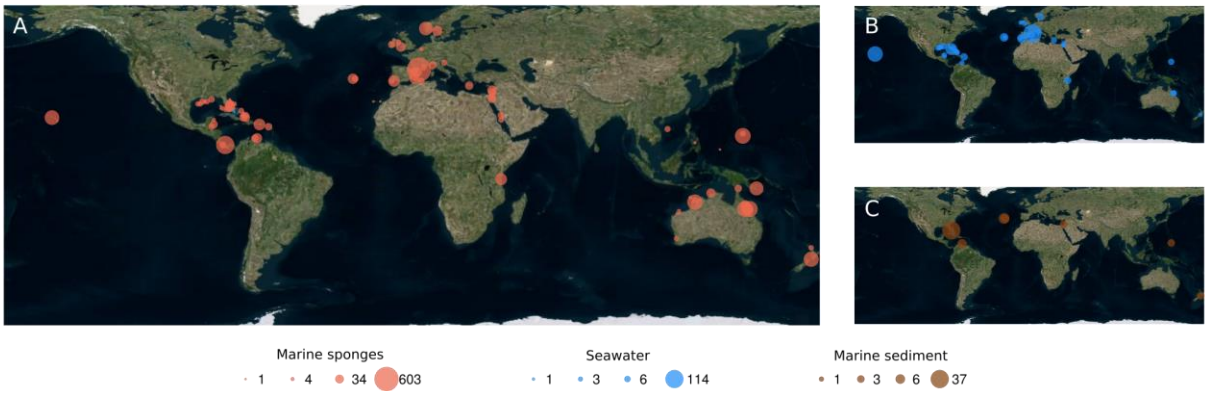


Figure 2.

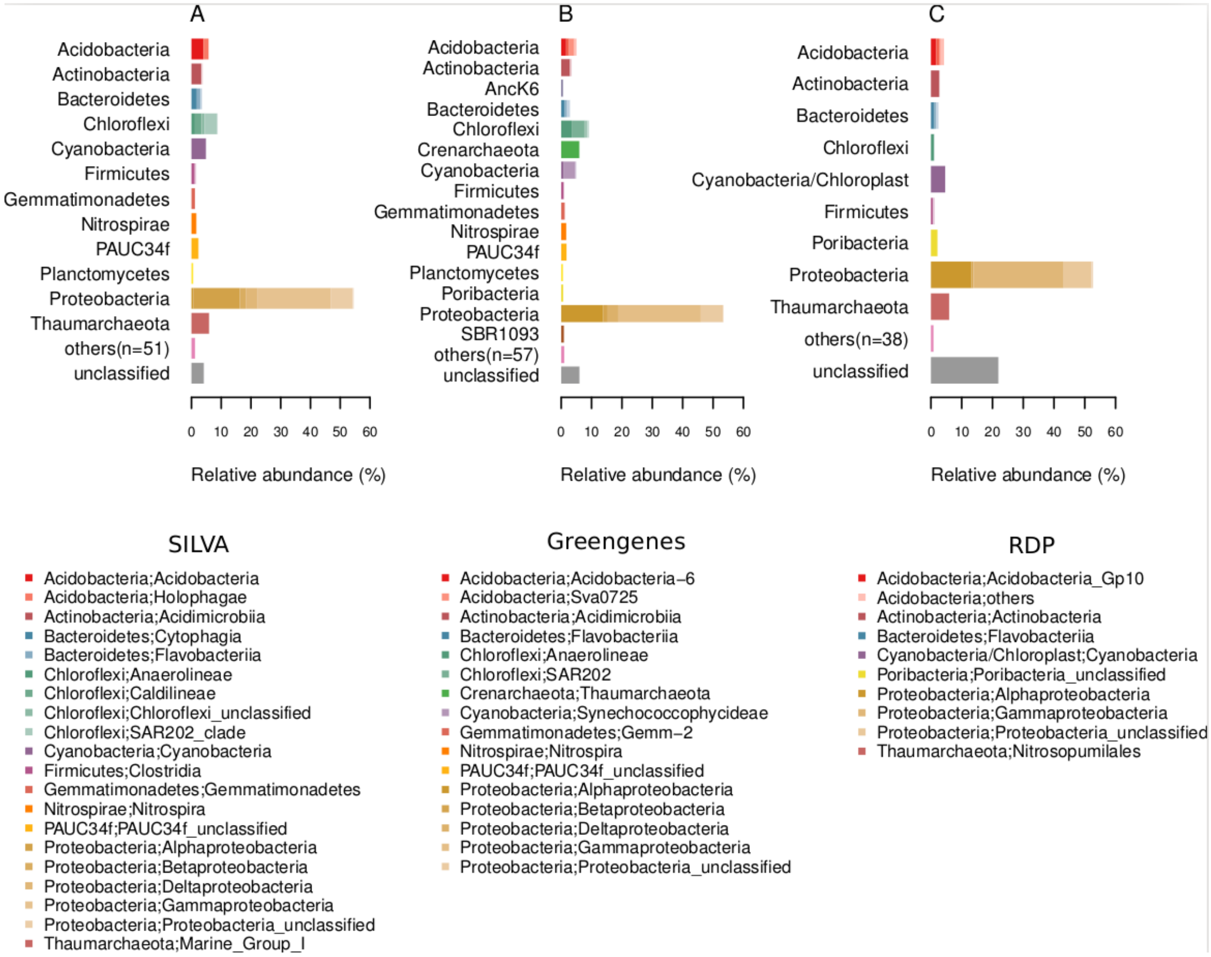
A
Acidobacteria
Actinobacteria
Bacteroidetes
Chloroflexi
Cyanobacteria
Firmicutes
Gemmatimonadetes
Nitrospirae
PAUC34f
Planctomycetes
Proteobacteria
Thaumarchaeota
others(n=51)
unclassified
0 10 20 30 40 50 60
Relative abundance (%)
B
Acidobacteria
Actinobacteria
AncK6
Bacteroidetes
Chloroflexi
Crenarchaeota
Cyanobacteria
Firmicutes
Gemmatimonadetes
Nitrospirae
PAUC34f
Planctomycetes
Poribacteria
Proteobacteria
SBR1093
others(n=57)
unclassified
0 10 20 30 40 50 60
Relative abundance (%)
C
Acidobacteria
Actinobacteria
Bacteroidetes
Chloroflexi
Cyanobacteria/Chloroplast
Firmicutes
Poribacteria
Proteobacteria
Thaumarchaeota
others(n=38)
unclassified
0 10 20 30 40 50 60
Relative abundance (%)
SILVA
Acidobacteria;Acidobacteria
Acidobacteria;Holophagae
Actinobacteria;Acidimicrobiia
Bacteroidetes;Cytophagia
Bacteroidetes;Flavobacteriia
Chloroflexi;Anaerolineae
Chloroflexi;Caldilineae
Chloroflexi;Chloroflexi_unclassified
Chloroflexi;SAR202_clade
Cyanobacteria;Cyanobacteria
Firmicutes;Clostridia
Gemmatimonadetes;Gemmatimonadetes
Nitrospirae;Nitrospira
PAUC34f;PAUC34f_unclassified
Proteobacteria;Alphaproteobacteria
Proteobacteria;Betaproteobacteria
Proteobacteria;Deltaproteobacteria
Proteobacteria;Gammaproteobacteria
Proteobacteria;Proteobacteria_unclassified
Thaumarchaeota;Marine_Group_I
Greengenes
Acidobacteria;Acidobacteria−6
Acidobacteria;Sva0725
Actinobacteria;Acidimicrobiia
Bacteroidetes;Flavobacteriia
Chloroflexi;Anaerolineae
Chloroflexi;SAR202
Crenarchaeota;Thaumarchaeota
Cyanobacteria;Synechococcophycideae
Gemmatimonadetes;Gemm−2
Nitrospirae;Nitrospira
PAUC34f;PAUC34f_unclassified
Proteobacteria;Alphaproteobacteria
Proteobacteria;Betaproteobacteria
Proteobacteria;Deltaproteobacteria
Proteobacteria;Gammaproteobacteria
Proteobacteria;Proteobacteria_unclassified
RDP
Acidobacteria;Acidobacteria_Gp10
Acidobacteria;others
Actinobacteria;Actinobacteria
Bacteroidetes;Flavobacteriia
Cyanobacteria/Chloroplast;Cyanobacteria
Poribacteria;Poribacteria_unclassified
Proteobacteria;Alphaproteobacteria
Proteobacteria;Gammaproteobacteria
Proteobacteria;Proteobacteria_unclassified
Thaumarchaeota;Nitrosopumilales

342 Figure 3.

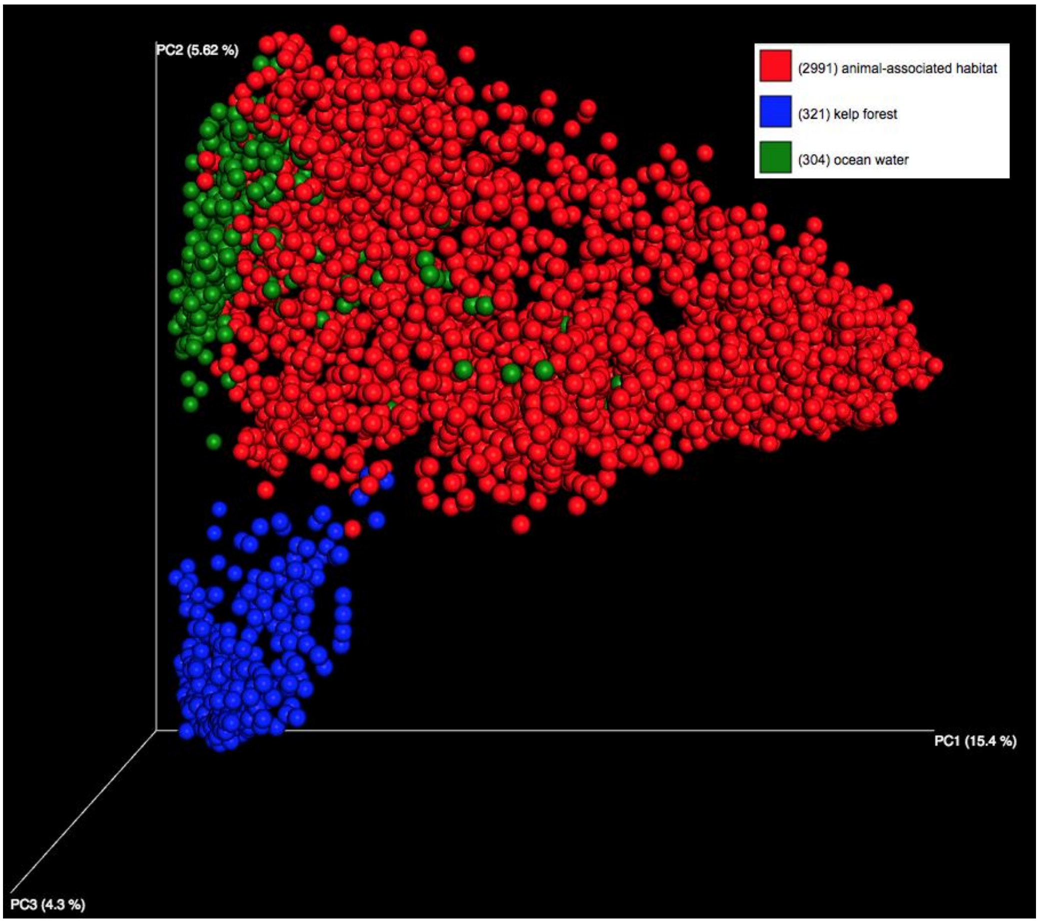

343

Figure 4.

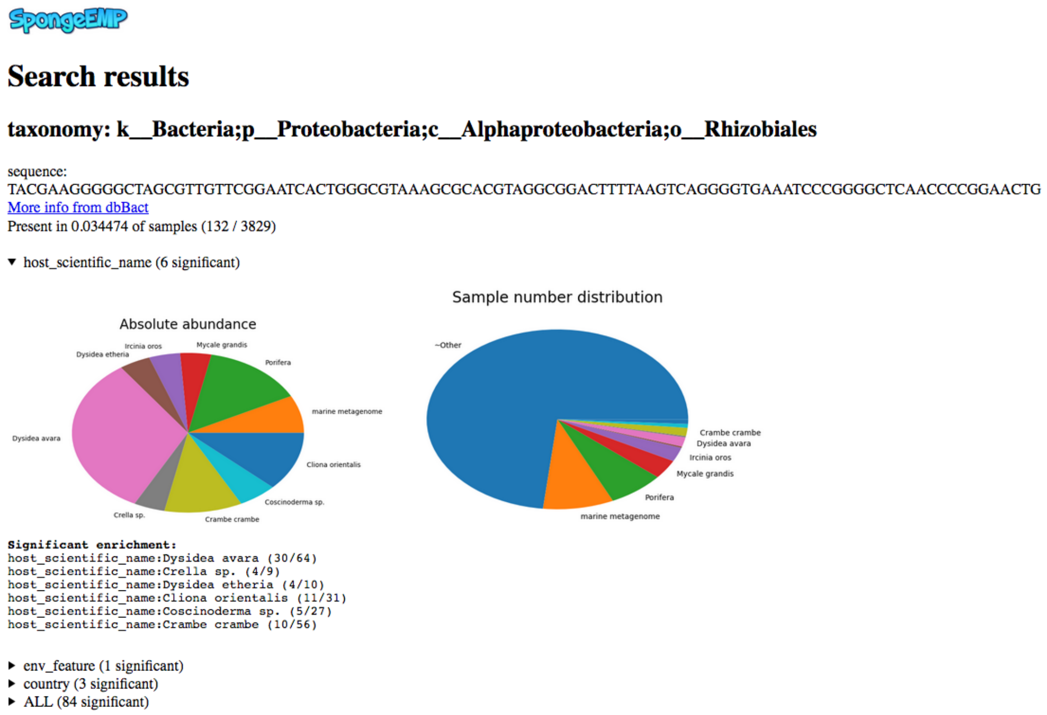


Legends

Figure 1. Global sample collection sites. Bubbles indicate collection sites of (A) marine sponges, (B) seawater and (C) marine sediment samples. Bubble sizes are proportional to number of samples as indicated.

Figure 2. Microbial taxonomic profile of marine sponge samples classified using (A) SILVA, (B) Greengenes and (C) RDP. OTU sequence counts were grouped according to phylum and class. Taxa with relative abundances ≤ 0.5% were grouped as 'others'. Classes with relative abundances > 1% are shown in the legend (phylum ";" class). Relative abundances are represented on the x-axes.

Figure 3. Unweighted UniFrac Principal Coordinates Analysis (PCA) of samples from sponges ("animal=associated", red), ocean water (green) and kelp (blue). A separation can be seen between samples based to the environmental origin. Samples were rarefying to 10,000 sequences per sample.

Figure 4. Output of the enrichment analysis through the online server www.spongeemp.com. Top line shows taxonomic assignment for the user-submitted sequence in the second line. Pie charts below show relative abundance and sample distribution plus their

associated significant enrichment results for the submitted sequence based on the scientific names of the host. At the bottom, fields can be opened to show results of the enrichment analyses for other metadata types (e.g. country).

Figure 1

Click here to download Figure Figure 1.png

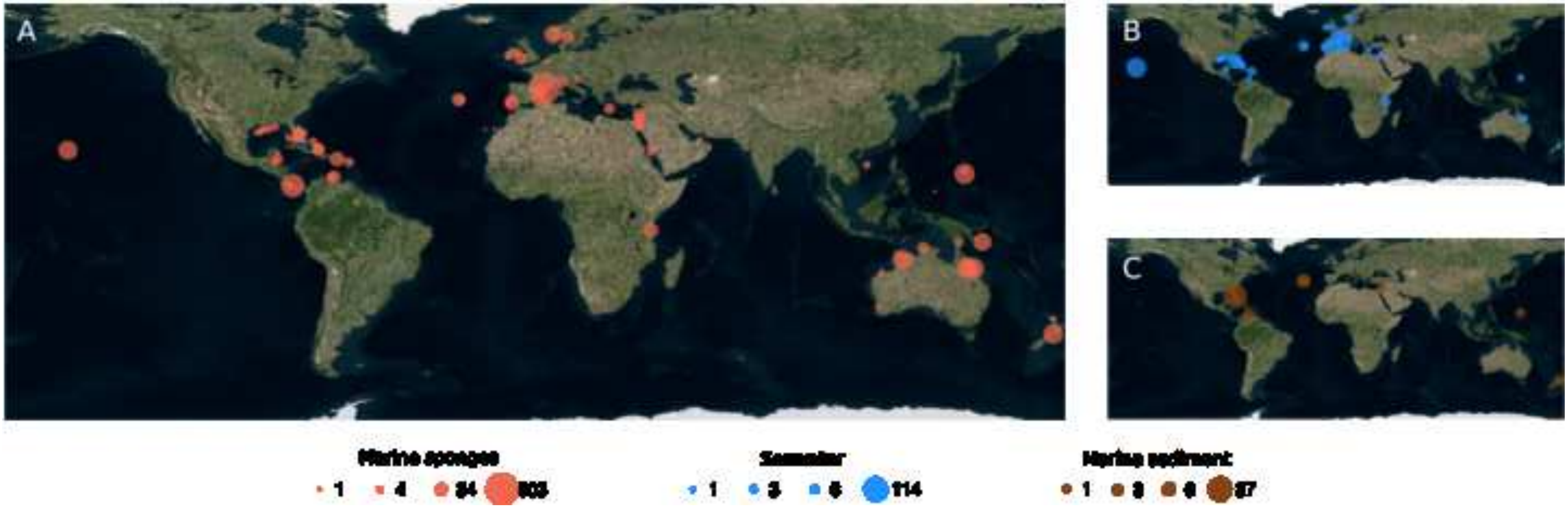

Figure 2

Click here to download Figure Figure 2.pdf

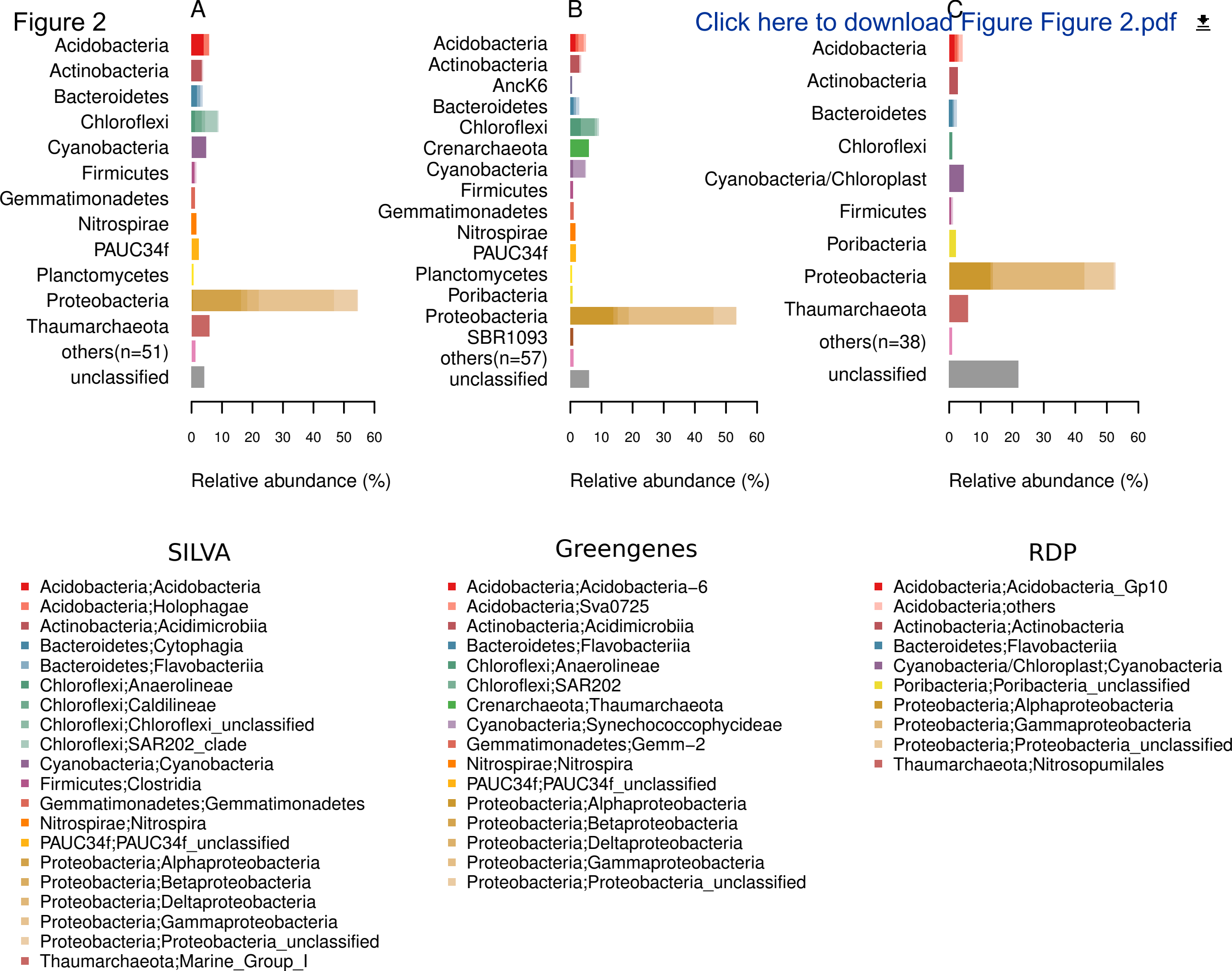

Figure 3

Click here to download Figure Figure 3.png

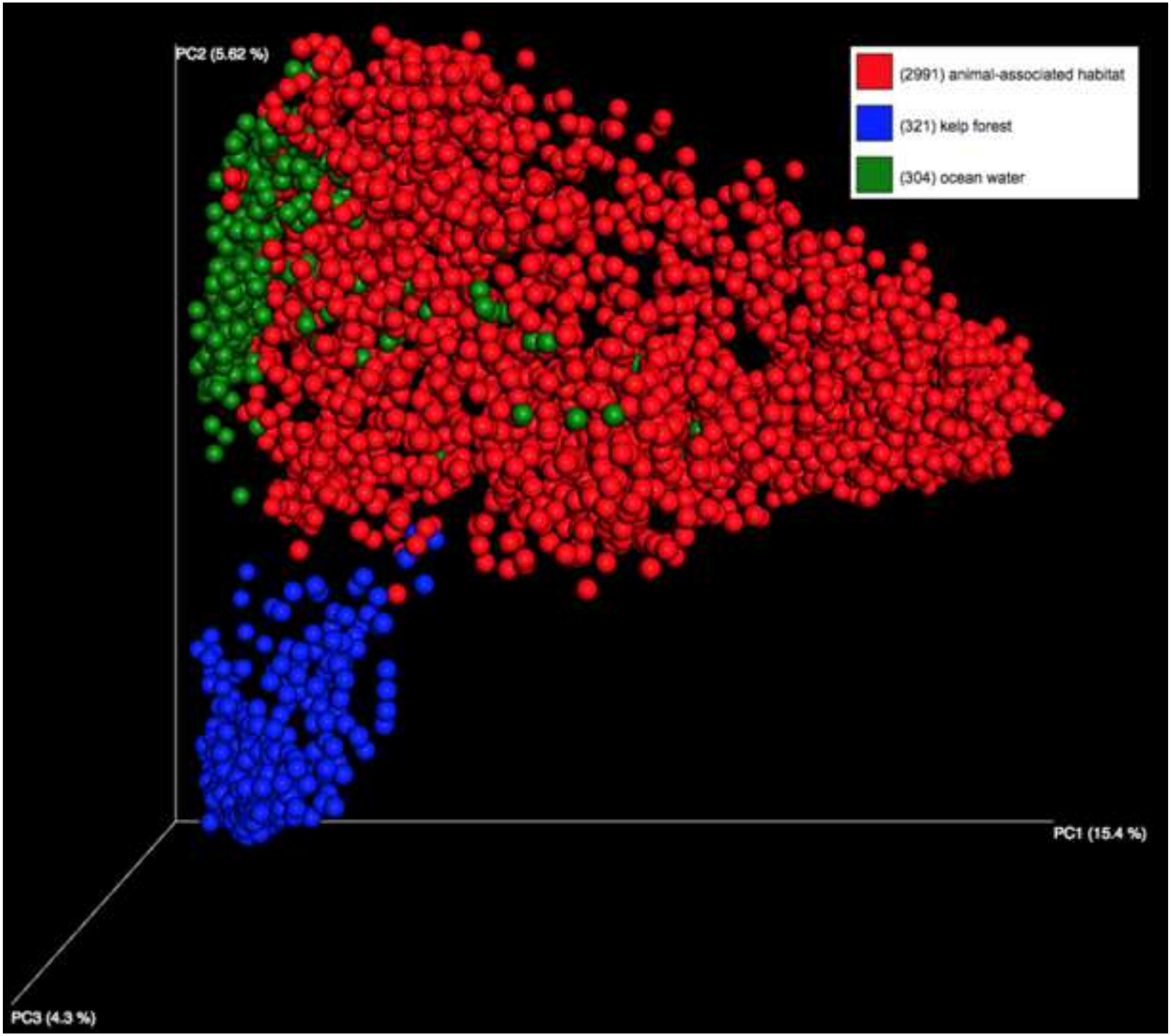

Figure 4

Click here to download Figure figure 4.pdf

# Search results

## taxonomy: k__Bacteria;p__Proteobacteria;c__Alphaproteobacteria;o__Rhizobiales

sequence:
TACGAAGGGGGCTAGCGTTGTTCGGAATCACTGGGCGTAAAGCGCACGTAGGCGGACTTTTAAGTCAGGGGTGAAATCCCGGGGCTCAACCCCGGAACTG
More info from dbBact
Present in 0.034474 of samples (132 / 3829)

▾ host_scientific_name (6 significant)

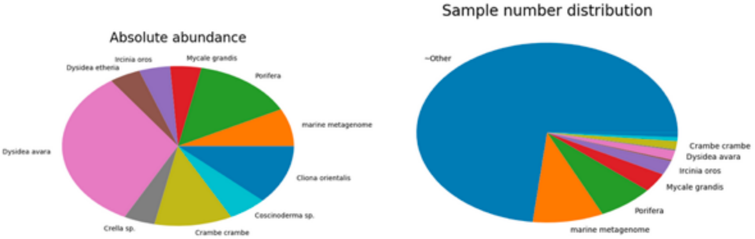


```
Significant enrichment:
host_scientific_name:Dysidea avara (30/64)
host_scientific_name:Crella sp. (4/9)
host_scientific_name:Dysidea etheria (4/10)
host_scientific_name:Cliona orientalis (11/31)
host_scientific_name:Coscinoderma sp. (5/27)
host_scientific_name:Crambe crambe (10/56)
```

▸ env_feature (1 significant)
▸ country (3 significant)
▸ ALL (84 significant)

Supplementary Table 1

Click here to access/download
**Supplementary Material**
Supp.table1.tsv

Data Information

Click here to access/download
**Supplementary Material**
Data information.v2.docx

README of files to be submitted

Click here to access/download
**Supplementary Material**
README.txt

Sequence processing notebook

Click here to access/download
**Supplementary Material**
SMP.sequence.processing.html

Sample metadata

Click here to access/download
**Supplementary Material**
sample.metadata.csv
