# Supplementary material for: The sponge microbiome project
Source: Gigascience. 2017 Aug 16;6(10):gix077. doi: 10.1093/gigascience/gix077 (PMC5632291; doi:10.1093/gigascience/gix077)
Supplement: GIGA-D-17-00079_Revision-1.pdf [file gix077_giga-d-17-00079_revision-1.pdf]

Manuscript with DOI
Click here to download Manuscript SMP.gigascience.revised_with DOI.docx

The sponge microbiome project

Lucas Moitinho-Silva[1], Shaun Nielsen[1], Amnon Amir[2], Antonio Gonzalez[2], Gail L. Ackermann[2], Carlo Cerrano[3], Carmen Astudillo-Garcia[4], Cole Easson[5], Detmer Sipkema[6], Fang Liu[7], Georg Steinert[6], Giorgos Kotoulas[7], Grace P. McCormack[8], Guofang Feng[9], James J. Bell[10], Jan Vicente[11], Johannes R Björk[12], Jose M. Montoya[13], Julie B. Olson[14], Julie Reveillaud[15], Laura Steindler[16], Mari-Carmen Pineda[17], Maria V. Marra[9], Micha Ilan[18], Michael W. Taylor[3], Paraskevi Polymenakou[8], Patrick M. Erwin[19], Peter J. Schupp[20], Rachel L. Simister[21], Rob Knight[2,22], Robert W. Thacker[23], Rodrigo Costa[24], Russell T. Hill[25], Susanna Lopez-Legentil[19], Thanos Dailianis[8], Timothy Ravasi[26], Ute Hentschel[27], Zhiyong Li[6], Nicole S. Webster[17, 28] and Torsten Thomas[1,*]

[1]Centre for Marine Bio-Innovation and School of Biological, Earth and Environmental Sciences, The University of New South Wales, Sydney, 2052, Australia

[2]Department of Pediatrics, University of California - San Diego, La Jolla, CA 92093, USA

[3]Department of Life and Environmental Sciences, Polytechnic University of Marche, Ancona, 60131, Italy

[4]School of Biological Sciences, University of Auckland, Auckland, New Zealand

[5]Halmos College of Natural Sciences and Oceanography, Nova Southeastern University, Dania Beach, FL 33004, USA

[6]Wageningen University, Laboratory of Microbiology, Stippeneng 4, 6708 WE Wageningen, The Netherlands

[7]Hellenic Centre for Marine Research, Institute of Marine Biology, Biotechnology and Aquaculture, Thalassocosmos 71500 Heraklion Greece

[8]Zoology, School of Natural Sciences, Ryan Institute, National University of Ireland Galway, University Rd., Galway, Ireland

[9]State Key Laboratory of Microbial Metabolism and School of Life Sciences and Biotechnology, Shanghai Jiao Tong University, Shanghai 200240, P.R. China

[10]School of Biological Sciences, Victoria University of Wellington, Wellington, New Zealand

[11]Hawaii Institute of Marine Biology, 46-007 Lilipuna Road, Kaneohe, HI 96744-1346

[12]Galvin Life Science Center, University of Notre Dame, Notre Dame, IN 46556, USA and Ecological Networks and Global Change Group, Theoretical and Experimental Ecology Station, CNRS, Moulis, France

[13]Ecological Networks and Global Change Group, Theoretical and Experimental Ecology Station, CNRS and Paul Sabatier University, Moulis, France

[14]Department of Biological Sciences, University of Alabama, Tuscaloosa, AL 35487, USA

[15]INRA, UMR1309 CMAEE; Cirad, UMR15 CMAEE, 34398 Montpellier, France

[16]Department of Marine Biology, Leon H. Charney School of Marine Sciences, University of Haifa, Haifa, Israel

[17]Australian Institute of Marine Science (AIMS), Townsville, 4810, Queensland, Australia

[18]Department of Zoology, George S. Wise Faculty of Life Sciences, Tel Aviv University, Tel Aviv 69978, Israel

[19]Department of Biology and Marine Biology, University of North Carolina Wilmington, Wilmington NC 28409, USA

[20]Institute for Chemistry and Biology of the Marine Environment (ICBM), Carl-von-Ossietzky and University Oldenburg, Schleusenstr. 1, 26382 Wilhelmshaven, Germany

[21]Department of microbiology and immunology, University of British Columbia, Canada, V6T 1Z3

[22]Department of Computer Science and Engineering, and Center for Microbiome Innovation, University of California - San Diego, La Jolla, CA 92093, USA

[23]Department of Ecology and Evolution, Stony Brook University, Stony Brook NY 11794, USA

[24]Institute for Bioengineering and Biosciences (IBB), Department of Bioengineering, IST, Universidade de Lisboa, Lisbon, Portugal

[25]Institute of Marine and Environmental Technology, University of Maryland Center for Environmental Science, 701 East Pratt Stree, Baltimore, MD 21202, USA

[26]KAUST Environmental Epigenetic Program (KEEP), Division of Biological and Environmental Sciences & Engineering, King Abdullah University of Science and Technology, Thuwal, Kingdom of Saudi Arabia

[27]RD3 Marine Microbiology, GEOMAR Helmholtz Centre for Ocean Research, Kiel, and Christian-Albrechts-University of Kiel, Germany

[28]Australian Centre for Ecogenomics, School of Chemistry and Molecular Biosciences, University of Queensland, St Lucia, QLD, Australia

*Corresponding author: t.thomas@unsw.edu.au; Centre for Marine Bio-Innovation, UNSW, Sydney, Australia

Abstract

Background: Marine sponges (phylum Porifera) are a diverse, phylogenetically deep-branching clade known for forming intimate partnerships with complex communities of microorganisms. To date, 16S rRNA gene sequencing studies have largely utilised different extraction and amplification methodologies to target the microbial communities of a limited number of sponge species, severely limiting comparative analyses of sponge microbial diversity and structure. Here, we provide an extensive and standardised dataset that will facilitate sponge microbiome comparisons across large spatial, temporal and environmental scales.

Findings: Samples from marine sponges (n=3569 specimens), seawater (n=370), marine sediments (n=65) and other environments (n=29) were collected from different locations across the globe. This dataset incorporates at least 269 different sponge species, including several yet unidentified taxa. The V4 region of the 16S rRNA gene was amplified and sequenced from extracted DNA using standardised procedures. Raw sequences (total of 1.1 billion sequences) were processed and clustered with a) a standard protocol using QIIME closed-reference picking resulting in 39,543 Operational Taxonomic Units (OTU) at 97% sequence identity, b) a *de novo* protocol using Mothur resulting in 518,246 OTUs, and c) a new high-resolution Deblur protocol resulting in 83,908 unique bacterial sequences. Abundance tables, representative sequences, taxonomic classifications and metadata are provided.

Conclusions: This dataset represents a comprehensive resource of sponge-associated microbial communities based on 16S rRNA gene sequences that can be used to address overarching hypotheses regarding host-associated prokaryotes, including host-specificity, convergent evolution, environmental drivers of microbiome structure and the sponge-associated rare biosphere.

Keywords: Marine sponges, Archaea, Bacteria, Symbiosis, Microbiome, 16S rRNA gene, Microbial diversity

Data Description

Purpose of data acquisition

Sponges (phylum Porifera) are an ancient metazoan clade [1], with more than 8,500 formally described species [2]. Sponges are benthic organisms that have important ecological functions in aquatic habitats [3, 4]. Marine sponges are often found in symbiotic association with microorganisms and these microbial communities can be very diverse and complex [5, 6]. Sponge symbionts perform a wide range of functional roles, including vitamin synthesis, production of bioactive compounds and biochemical transformations of nutrients or waste products [7-9]. The diversity of microorganisms associated with sponges has been the subject of intense study (the search of "sponge microbial diversity" returned 348 publications in Scopus database [10]. Most of these studies were performed on individual species from restricted geographic regions [e.g., 11, 12]. A comparative assessment of these studies is often hindered by differences in sample processing and 16S rRNA gene sequencing. However, two recent studies incorporating a large number of sponge microbiomes (> 30) [5, 13] revealed the potential of large-scale, standardised, high-throughput sequencing for gaining insights into the diversity and structure of sponge-associated microbial communities. The purpose of this global dataset is to provide a comprehensive 16S rRNA gene-based resource for investigating and comparing microbiomes more generally across the phylum Porifera.

Sample collection, processing and 16S rRNA gene sequencing

Sample collection and processing, species identification and DNA extractions were conducted as previously described [13]. A total of 3569 sponge specimens were collected, representing at least 268 species, including several yet unidentified taxa (hereafter collectively referred to as species) (Supplementary Table S1). Of the total species, 213 were represented by at least three specimens. *Carteriospongia foliascens* had the highest replication comprising 150 individuals. Seawater (n=370), sediment (n=65), algae (n=1) and echinoderm (n=1) samples as well as biofilm swabs (n=21) of rock surfaces were collected in close proximity to the sponges for comparative community analysis. Six negative control samples (sterile water) were processed to identify any potential contaminations. Of the samples included in this current dataset, 973 samples had been analysed previously [13]. Samples were collected from a wide range of geographical locations (Figure 1 and Supplementary Table S1). Total DNA was extracted as previously described [13] and used as templates to amplify and sequence the V4 region of the 16S rRNA gene using the standard procedures of the EMP [14, 15].

Processing of sequencing data

Clustering using the EMP standard protocols in QIIME:

Raw sequences were demultiplexed and quality controlled following the recommendations of [16]. Quality-filtered, demultiplexed fastq files were processed using the default closed-reference pipeline from QIIME v. 1.9.1 (QIIME, RRID:SCR_008249). Briefly, sequences were matched against GreenGenes reference database (v. 13_8 clustered at 97% similarity). Sequences that failed to align (e.g. chimeras) were discard, which resulted in a final number of 300,140,110 sequences. Taxonomy assignments and the phylogenetic tree information were taken from the centroids of the reference sequence clusters contain in the GreenGenes reference database (Greengenes, RRID:SCR_002830). This closed-reference analysis allows for cross-dataset comparisons and direct comparison with the tens of thousands of other samples processed in the EMP and available via the Qiita database [17].

Clustering using Mothur:

Quality-filtered, demultiplexed fastq files were also processed using Mothur v. 1.37.6 (mothur, RRID:SCR_011947) [18] and Python v. 2.7 (Python Programming Language, RRID:SCR_008394) [19] custom scripts with modifications from previously established protocols [13]. Detailed descriptions and command outputs are available at the project notebook (see Availability of supporting data). Briefly, sequences were quality-trimmed to a maximum length of 100 bp. To minimize computational effort, the dataset was reduced to unique sequences, retaining total sequence counts. Sequences were aligned to the V4 region of the 16S rRNA gene sequences from the SILVA v. 123 database (SILVA, RRID: SCR_006423) [20]. Sequences that aligned at the expected positions were kept and this dataset was again reduced to unique sequences. Further, singletons were removed from the dataset and remaining sequences were pre-clustered if they differed by one nucleotide position. Sequences classified as eukaryote, chloroplast, mitochondria or unknown according to the Greengenes (v. 13_8 clustered at 99% similarity) [21] and SILVA taxonomies [22] were removed. Chimeras were identified with UCHIME (UCHIME, RRID: SCR_008057) [23] and removed. Finally, sequences were *de novo* clustered into Operational Taxonomic Units (OTUs) using the furthest neighbour method at 97% similarity. Representative sequences of OTUs were retrieved based on the mean distance among the clustered sequences. Consensus taxonomies based on the SILVA, Greengenes and RDP (v. 14_032015; Ribosomal Database Project, RRID: SCR_006633) [24] databases were obtained based on the classification of sequences clustered within each OTU. The inclusion of these taxonomies is helpful considering that they have substantial differences as recently discussed [25]. For example, Greengenes and RDP have the taxon Poribacteria, a prominent sponge-enriched phylum [26], which did not exist in the SILVA version used.

De-noising using Deblur:

Recently, sub-OTU methods that allow views of the data at single-nucleotide resolution have become available. One such method is Deblur [27], which is a denoising algorithm for identification of actual bacterial sequences present in a sample. Using an upper bound on the PCR and read-error rates, Deblur processes each sample independently and outputs the list of sequences and their frequencies in each sample, enabling single nucleotide resolution. For creating the deblurred biom table, quality filtered, demultiplexed fasta files were used as input to Deblur using a trim length of 100, and min-reads of 25 (removing sOTUs with < 25 reads total in all samples combined). Taxonomy was added to resulting biom table using QIIME [28], RDP classifier [29] and Greengenes v. 13.8 [21].

Database metadata category enrichment:

For enrichment analysis of metadata terms in a set of sequences, each unique metadata value is tested using both a binomial test and a ranksum test. All analysis is performed on a randomly subsampled (to 5000 reads/sample) table.

The binomial (presence/absence) p-value for enrichment calculated as follows:

For a bacterial sequence s and metadata value v, denote N the total number of samples, O(s) the number of samples where s is present, $K_v(s)$ the number of sample with value v where s is present, and T(v) the total number of samples with value v.

p-value = *binomial_cdf* ($T(v)-K_v(s)$, T(v), $P_{Null}(s)$)

where $P_{Null}(s) = O(s) / N$

The ranksum (frequency aware) p-value is calculated using the Kruskal-Wallis test (implemented in scipy 0.19) as follows:

For a bacterial sequence s and metadata value v, denote by $F_v(s)$ the vector of relative frequencies of bacteria s in all samples with metadata value v, and denote by $\widehat{F_v(s)}$ the vector of relative frequencies of bacteria s in all samples with metadata other than v. The ranksum p-value is then calculated using the Kruskal-Wallis test for $F_v(s)$ and $\widehat{F_v(s)}$, and shown only if significantly enriched in samples containing v (i.e. rank difference of $F_v(s)$ - $\widehat{F_v(s)}$ > 0).

We have set up a webserver (www.spongeemp.com) that performs this enrichment analysis for user-defined sequence submissions. The code for the webserver is also available in Github [29] for a local installation.

Data description

The dataset covers 4033 samples with a total of 1,167,226,701 raw sequence reads. These sequence reads clustered into 39,543 OTUs using QIIME's closed-reference processing, 518,246 OTUs from *de novo* clustering using Mothur (not filtered for OTU abundances), and 83,908 sOTUs using Deblur (with a filtering of at least 25 reads total per sOTU). We recommend that data users consider the differences in sequencing depths per sample and abundance filtering for certain downstream analyses, such as when calculating diversity estimates [16] and comparing OTU abundances across samples [31]. In terms of taxonomic diversity, most Mothur OTUs were assigned to the phylum Proteobacteria, although more than 60 different microbial phyla were recovered from the marine sponge samples according to SILVA (n=63) and Greengenes classifications (n=72) (Figure 2).

Potential uses

This dataset can be utilised to assess a broad range of ecological questions pertaining to host-associated microbial communities generally or to sponge microbiology specifically. These include: i) the degree of host-specificity, ii) the existence of biogeographic or environmental patterns, iii) the relation of microbiomes to host phylogeny, iv) the variability of microbiomes within or between host species, v) symbiont co-occurrence patterns as well as vi) assessing the existence of a core sponge microbiome. An example of this type of analysis is shown in Figure 3, where samples were clustered using unweighted UniFrac data [10] with a Principal Coordinate Analysis and visualization in Emperor [15] based on their origins from sponges, seawater or kelps [17].

Availability and requirements

Project name: The Sponge Microbiome Project

Project home page: www.spongeemp.com; https://github.com/amnona/SpongeEMP

Operating system(s): Unix

Programming language: Python and R

Other requirements: Python v. 2.7, Biopython v. 1.65, Python 3.5, R v. 3.2.2, Mothur v. 1.37.6, QIIME v. 1.9.1, Deblur

License: MIT

Any restrictions to use by non-academics: None

Availability of supporting data

Raw sequence data were deposited in the European Nucleotide Archive (accession numbers: ERP020690). Quality-filtered, demultiplexed fastq files, Deblur and QIIME resulting OTU tables are available at Qiita database [17] (Study ID: 10793). The additional datasets that support the results of this article are available in the GigaScience repository, GigaDB [32] and include an OTU abundance matrix (the output ".shared" file from Mothur, which is tab delimited), an OTU taxonomic classification table (tab delimited text file), an OTU representative sequence FASTA file, a table of samples' metadata, the biom file of the QIIME analysis and the associated tree file. The project workflow, Mothur commands and additional scripts are available as HTML in GigaDB [32], which is viewed in any browser.

The deblurred dataset has also been uploaded to an online server [19] that supplies both html and REST-API access for querying bacterial sequences and obtaining the observed prevalence and enriched metadata categories where the sequence is observed (Figure 4). This allows an interactive view of which sequences are associated with which specific parameters, such as depth or salinity.

List of abbreviations

bp: base pairs

OTU: operational taxonomic unit

rRNA: ribosomal RNA

Competing interests

The authors declare that they have no competing interests.

Funding

T.T. and N.S.W were funded by an Australian Research Council Future Fellowship FT140100197 and FT120100480, respectively. T.T. received funds from the Gordon and Betty Moore Foundation. This work was also supported in part by the W.M. Keck Foundation and the John Templeton Foundation. R.K. received funding as a Howard Hughes Medical Institute Early Career Scientist.

Authors' contributions

L.M.-S., N.S.W. and T.T. designed the study. C.A.G., D.S., F.L., G.S., G.K., G.McC., G.-F. F, J.J.B., J.V., J.R.B., J.M.M., J.R., L.S., M.C.P, M.V.M., M.W.T., N.S.W., P.P., P.M.E., P.J.S., R.L.S, R.W.T., R.C., R.T.H., S.L-L., T.D., T.R., U.H. and Z-Y. L. collected samples. C.A.G., D.S., J.V., J.R.B., L.S., M.C.P., M.W.T., N.S.W., P.M.E., R.L.S, R.W.T., S.L-L. and U.H. extracted DNA. G.L.A. and R.K. sequenced DNA. L.M.-S., S.N., A.A., A.G., G.L.A. and T.T. performed data processing and analysis. L.M.-S., N.S.W. and T.T. wrote the manuscript. All authors contributed to the writing of the manuscript.

References

1. Li CW, Chen JY and Hua TE. Precambrian sponges with cellular structures. Science. 1998;279 5352:879-82.
2. Van Soest RW, Boury-Esnault N, Vacelet J, Dohrmann M, Erpenbeck D, De Voogd NJ, et al. Global diversity of sponges (Porifera). PLoS One. 2012;7 4:e35105. doi:10.1371/journal.pone.0035105.
3. Bell JJ. The functional roles of marine sponges. Estuar Coast Shelf S. 2008;79 3:341-53.
4. de Goeij JM, van Oevelen D, Vermeij MJ, Osinga R, Middelburg JJ, de Goeij AF, et al. Surviving in a marine desert: the sponge loop retains resources within coral reefs. Science. 2013;342 6154:108-10. doi:10.1126/science.1241981.
5. Schmitt S, Tsai P, Bell J, Fromont J, Ilan M, Lindquist N, et al. Assessing the complex sponge microbiota: core, variable and species-specific bacterial communities in marine sponges. ISME J. 2012;6 3:564-76. doi:10.1038/ismej.2011.116.
6. Webster NS, Taylor MW, Behnam F, Lucker S, Rattei T, Whalan S, et al. Deep sequencing reveals exceptional diversity and modes of transmission for bacterial sponge symbionts. Environ Microbiol. 2010;12 8:2070-82. doi:10.1111/j.1462-2920.2009.02065.x.
7. Siegl A, Kamke J, Hochmuth T, Piel J, Richter M, Liang C, et al. Single-cell genomics reveals the lifestyle of Poribacteria, a candidate phylum symbiotically associated with marine sponges. ISME J. 2011;5 1:61-70. doi:10.1038/ismej.2010.95.
8. Taylor MW, Radax R, Steger D and Wagner M. Sponge-associated microorganisms: evolution, ecology, and biotechnological potential. Microbiol Mol Biol Rev. 2007;71 2:295-347. doi:10.1128/MMBR.00040-06.

9. Wilson MC, Mori T, Ruckert C, Uria AR, Helf MJ, Takada K, et al. An environmental bacterial taxon with a large and distinct metabolic repertoire. Nature. 2014;506 7486:58-62. doi:10.1038/nature12959.
10. Lozupone C and Knight R. UniFrac: a new phylogenetic method for comparing microbial communities. Appl Environ Microbiol. 2005;71 12:8228-35. doi:10.1128/AEM.71.12.8228-8235.2005.
11. Moitinho-Silva L, Bayer K, Cannistraci CV, Giles EC, Ryu T, Seridi L, et al. Specificity and transcriptional activity of microbiota associated with low and high microbial abundance sponges from the Red Sea. Mol Ecol. 2014;23 6:1348-63. doi:10.1111/mec.12365.
12. Montalvo NF and Hill RT. Sponge-associated bacteria are strictly maintained in two closely related but geographically distant sponge hosts. Appl Environ Microbiol. 2011;77 20:7207-16. doi:10.1128/AEM.05285-11.
13. Thomas T, Moitinho-Silva L, Lurgi M, Bjork JR, Easson C, Astudillo-Garcia C, et al. Diversity, structure and convergent evolution of the global sponge microbiome. Nat Commun. 2016;7:11870. doi:10.1038/ncomms11870.
14. Gilbert JA, Jansson JK and Knight R. The Earth Microbiome project: successes and aspirations. BMC Biol. 2014;12:69. doi:10.1186/s12915-014-0069-1.
15. Vazquez-Baeza Y, Pirrung M, Gonzalez A and Knight R. EMPeror: a tool for visualizing high-throughput microbial community data. Gigascience. 2013;2 1:16. doi:10.1186/2047-217X-2-16.
16. Bokulich NA, Subramanian S, Faith JJ, Gevers D, Gordon JI, Knight R, et al. Quality-filtering vastly improves diversity estimates from Illumina amplicon sequencing. Nat Methods. 2013;10 1:57-9. doi:10.1038/nmeth.2276.
17. Marzinelli EM, Campbell AH, Zozaya Valdes E, Verges A, Nielsen S, Wernberg T, et al. Continental-scale variation in seaweed host-associated bacterial communities is a function of host condition, not geography. Environ Microbiol. 2015;17 10:4078-88. doi:10.1111/1462-2920.12972.
18. Schloss PD, Westcott SL, Ryabin T, Hall JR, Hartmann M, Hollister EB, et al. Introducing mothur: open-source, platform-independent, community-supported software for describing and comparing microbial communities. Appl Environ Microbiol. 2009;75 23:7537-41. doi:10.1128/AEM.01541-09.
19. Sponge microbiome project deblurred dataset online server. http://www.spongeemp.com. Accessed 31 Mar 2017.
20. Quast C, Pruesse E, Yilmaz P, Gerken J, Schweer T, Yarza P, et al. The SILVA ribosomal RNA gene database project: improved data processing and web-based tools. Nucleic Acids Res. 2013;41 Database issue:D590-6. doi:10.1093/nar/gks1219.
21. DeSantis TZ, Hugenholtz P, Larsen N, Rojas M, Brodie EL, Keller K, et al. Greengenes, a chimera-checked 16S rRNA gene database and workbench compatible with ARB. Appl Environ Microbiol. 2006;72 7:5069-72. doi:10.1128/AEM.03006-05.
22. Yilmaz P, Parfrey LW, Yarza P, Gerken J, Pruesse E, Quast C, et al. The SILVA and "All-species Living Tree Project (LTP)" taxonomic frameworks. Nucleic Acids Res. 2014;42 Database issue:D643-8. doi:10.1093/nar/gkt1209.
23. Edgar RC, Haas BJ, Clemente JC, Quince C and Knight R. UCHIME improves sensitivity and speed of chimera detection. Bioinformatics. 2011;27 16:2194-200. doi:10.1093/bioinformatics/btr381.
24. Cole JR, Wang Q, Fish JA, Chai B, McGarrell DM, Sun Y, et al. Ribosomal Database Project: data and tools for high throughput rRNA analysis. Nucleic Acids Res. 2014;42 Database issue:D633-42. doi:10.1093/nar/gkt1244.
25. Balvociute M and Huson DH. SILVA, RDP, Greengenes, NCBI and OTT - how do these taxonomies compare? BMC Genomics. 2017;18 Suppl 2:114. doi:10.1186/s12864-017-3501-4.

26. Fieseler L, Horn M, Wagner M and Hentschel U. Discovery of the novel candidate phylum "Poribacteria" in marine sponges. Appl Environ Microbiol. 2004;70 6:3724-32. doi:10.1128/AEM.70.6.3724-3732.2004.
27. Amir A, McDonald D, Navas-Molina JA, Kopylova E, Morton JT, Zech Xu Z, et al. Deblur rapidly resolves single-nucleotide community sequence patterns. mSystems. 2017;2 2 doi:10.1128/mSystems.00191-16.
28. Caporaso JG, Kuczynski J, Stombaugh J, Bittinger K, Bushman FD, Costello EK, et al. QIIME allows analysis of high-throughput community sequencing data. Nat Methods. 2010;7 5:335-6. doi:10.1038/nmeth.f.303.
29. Wang Q, Garrity GM, Tiedje JM and Cole JR. Naive Bayesian classifier for rapid assignment of rRNA sequences into the new bacterial taxonomy. Appl Environ Microbiol. 2007;73 16:5261-7. doi:10.1128/AEM.00062-07.
30. SpongeEMP GitHub. https://github.com/amnona/SpongeEMP. Accessed 31 Mar 2017.
31. McMurdie PJ and Holmes S. Waste not, want not: why rarefying microbiome data is inadmissible. PLoS Comput Biol. 2014;10 4:e1003531. doi:10.1371/journal.pcbi.1003531.32.
32. Moitinho-Silva L, Nielsen S, Amir A, Gonzalez A, Ackermann GL, Cerrano C et al. Supporting data for "The sponge microbiome project" GigaScience Database. 2017. http://dx.doi.org/10.5524/100332

Figures

Figure 1.

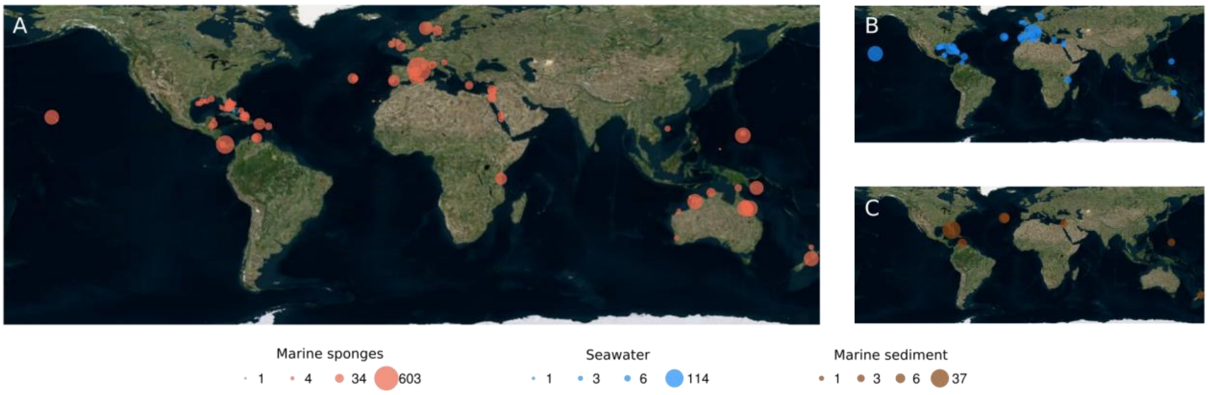

Figure 2.

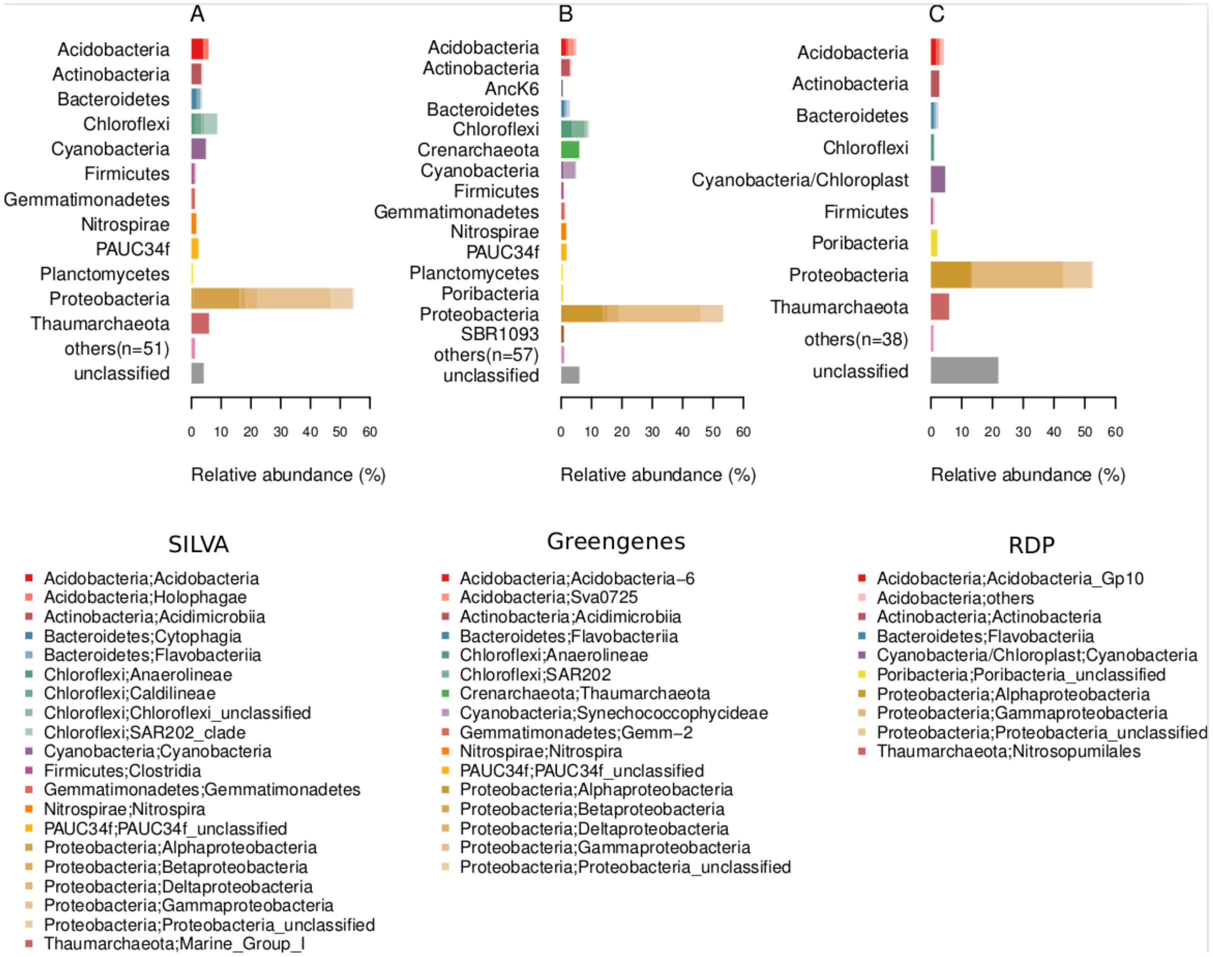
A
Acidobacteria
Actinobacteria
Bacteroidetes
Chloroflexi
Cyanobacteria
Firmicutes
Gemmatimonadetes
Nitrospirae
PAUC34f
Planctomycetes
Proteobacteria
Thaumarchaeota
others(n=51)
unclassified
0 10 20 30 40 50 60
Relative abundance (%)
B
Acidobacteria
Actinobacteria
AncK6
Bacteroidetes
Chloroflexi
Crenarchaeota
Cyanobacteria
Firmicutes
Gemmatimonadetes
Nitrospirae
PAUC34f
Planctomycetes
Poribacteria
Proteobacteria
SBR1093
others(n=57)
unclassified
0 10 20 30 40 50 60
Relative abundance (%)
C
Acidobacteria
Actinobacteria
Bacteroidetes
Chloroflexi
Cyanobacteria/Chloroplast
Firmicutes
Poribacteria
Proteobacteria
Thaumarchaeota
others(n=38)
unclassified
0 10 20 30 40 50 60
Relative abundance (%)
SILVA
Acidobacteria;Acidobacteria
Acidobacteria;Holophagae
Actinobacteria;Acidimicrobiia
Bacteroidetes;Cytophagia
Bacteroidetes;Flavobacteriia
Chloroflexi;Anaerolineae
Chloroflexi;Caldilineae
Chloroflexi;Chloroflexi_unclassified
Chloroflexi;SAR202_clade
Cyanobacteria;Cyanobacteria
Firmicutes;Clostridia
Gemmatimonadetes;Gemmatimonadetes
Nitrospirae;Nitrospira
PAUC34f;PAUC34f_unclassified
Proteobacteria;Alphaproteobacteria
Proteobacteria;Betaproteobacteria
Proteobacteria;Deltaproteobacteria
Proteobacteria;Gammaproteobacteria
Proteobacteria;Proteobacteria_unclassified
Thaumarchaeota;Marine_Group_I
Greengenes
Acidobacteria;Acidobacteria-6
Acidobacteria;Sva0725
Actinobacteria;Acidimicrobiia
Bacteroidetes;Flavobacteriia
Chloroflexi;Anaerolineae
Chloroflexi;SAR202
Crenarchaeota;Thaumarchaeota
Cyanobacteria;Synechococcophycideae
Gemmatimonadetes;Gemm-2
Nitrospirae;Nitrospira
PAUC34f;PAUC34f_unclassified
Proteobacteria;Alphaproteobacteria
Proteobacteria;Betaproteobacteria
Proteobacteria;Deltaproteobacteria
Proteobacteria;Gammaproteobacteria
Proteobacteria;Proteobacteria_unclassified
RDP
Acidobacteria;Acidobacteria_Gp10
Acidobacteria;others
Actinobacteria;Actinobacteria
Bacteroidetes;Flavobacteriia
Cyanobacteria/Chloroplast;Cyanobacteria
Poribacteria;Poribacteria_unclassified
Proteobacteria;Alphaproteobacteria
Proteobacteria;Gammaproteobacteria
Proteobacteria;Proteobacteria_unclassified
Thaumarchaeota;Nitrosopumilales

Figure 3.

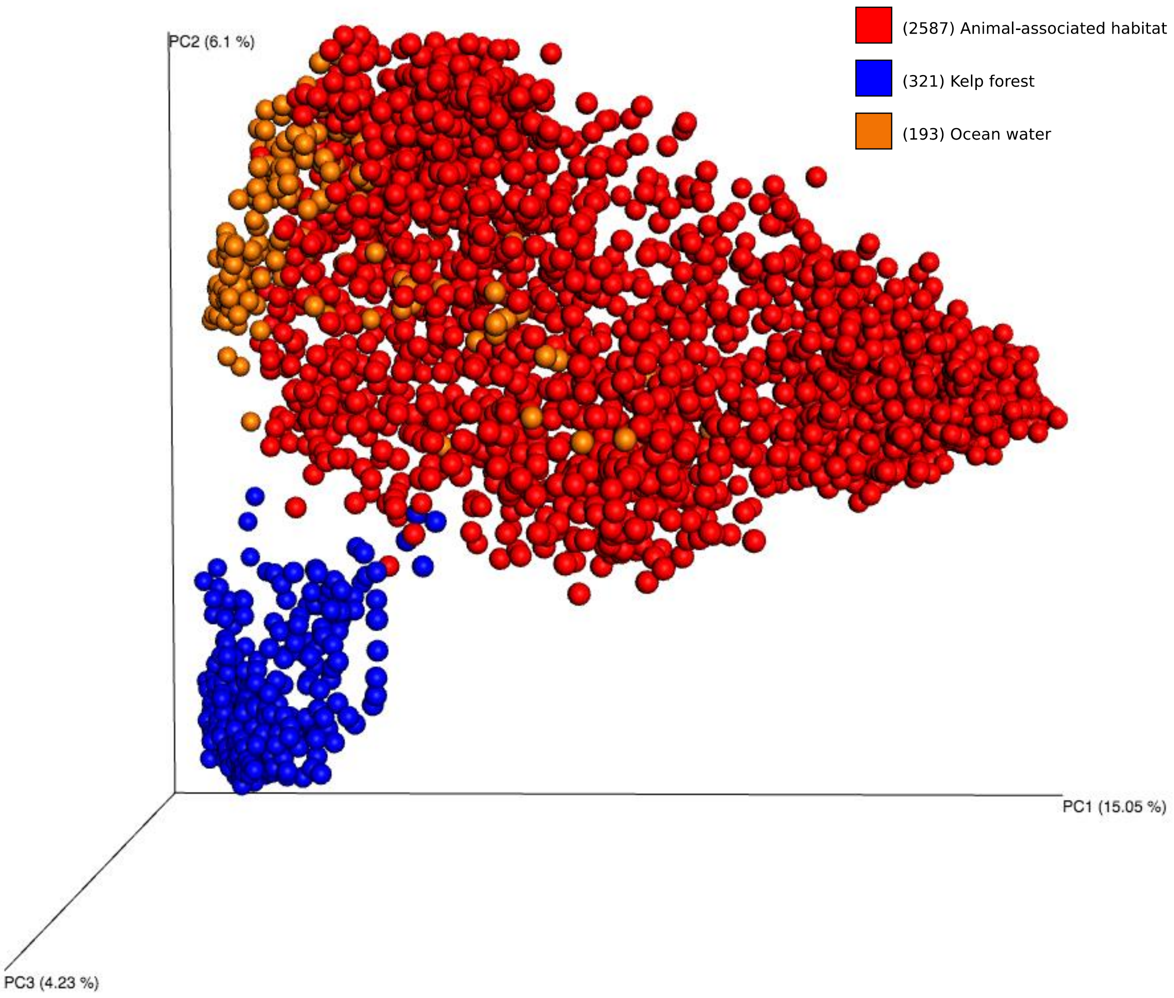
PC2 (6.1 %)
PC1 (15.05 %)
PC3 (4.23 %)
(2587) Animal-associated habitat
(321) Kelp forest
(193) Ocean water

Figure 4.

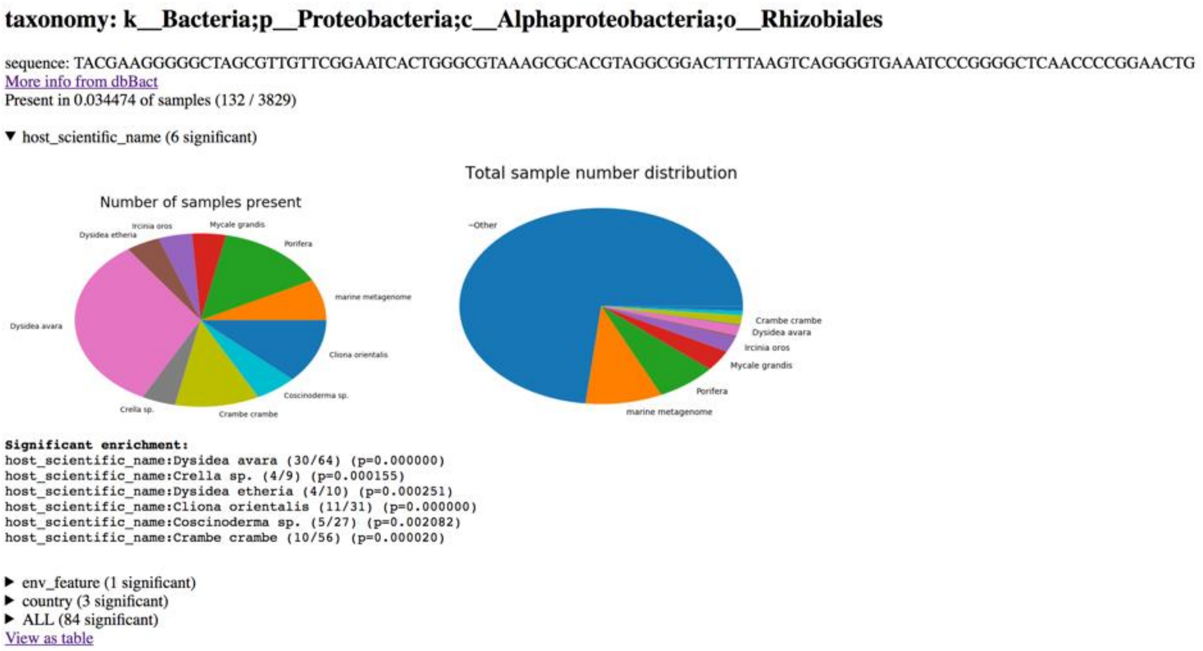

Legends

Figure 1. Global sample collection sites. Bubbles indicate collection sites of (A) marine sponges, (B) seawater and (C) marine sediment samples. Bubble sizes are proportional to number of samples as indicated.

Figure 2. Microbial taxonomic profile of marine sponge samples processed with Mothur. (A) SILVA, (B) Greengenes and (C) RDP taxonomies are shown. OTU sequence counts were grouped according to phylum and class. Taxa with relative abundances ≤ 0.5% were grouped as 'others'. Classes with relative abundances > 1% are shown in the legend (phylum ";" class). Relative abundances are represented on the x-axes.

Figure 3. Unweighted UniFrac Principal Coordinates Analysis (PCA) of samples from sponges ("animal-associated habitat"), kelp forest and ocean water. A separation can be seen between samples based to the environmental origin. Samples were rarefying to 10,000 sequences per sample. A movie showing the PCA plot in 3 D is provided in the supporting information.

Figure 4. Output of the enrichment analysis through the online server www.spongeemp.com. Top line shows taxonomic assignment for the user-submitted sequence in

the second line. Pie charts below show the total number of samples (right) and the number of samples where the submitted sequence is present (left) based on the scientific names of the host, followed by the significantly enriched host names containing the submitted sequence (using either presence/absence binomial test or relative-frequency based ranksum test). At the bottom, fields can be opened to show results of the enrichment analyses for other metadata types (e.g. country).

Figure 1 Click here to download Figure Figure 1.png

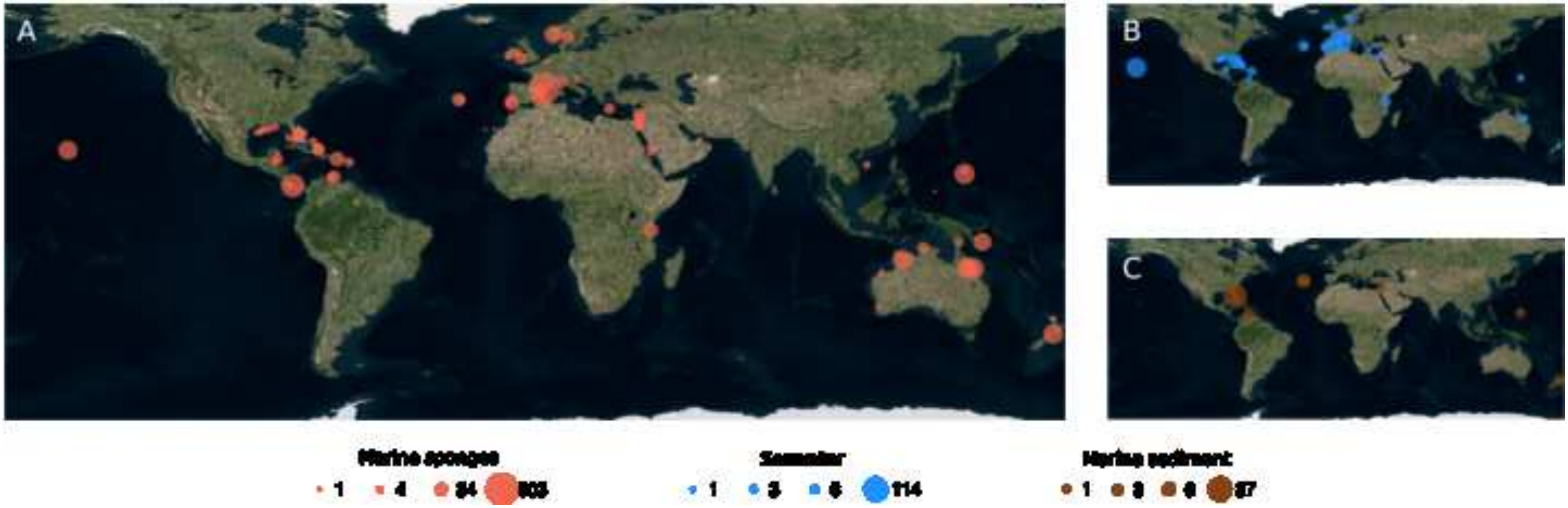

Figure 2

Click here to download Figure Figure 2.pdf

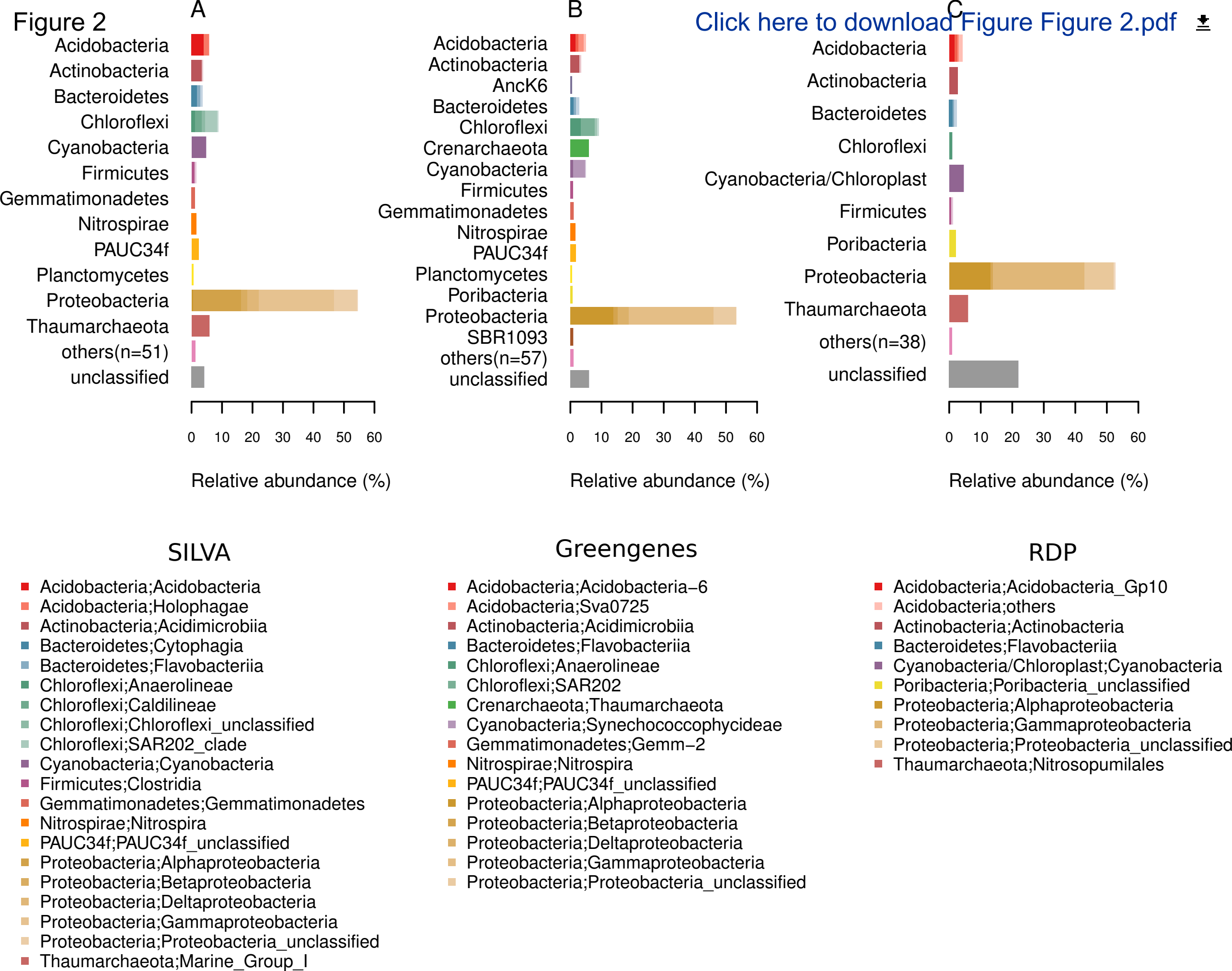

Figure 3

Click here to download Figure Figure.3.pdf

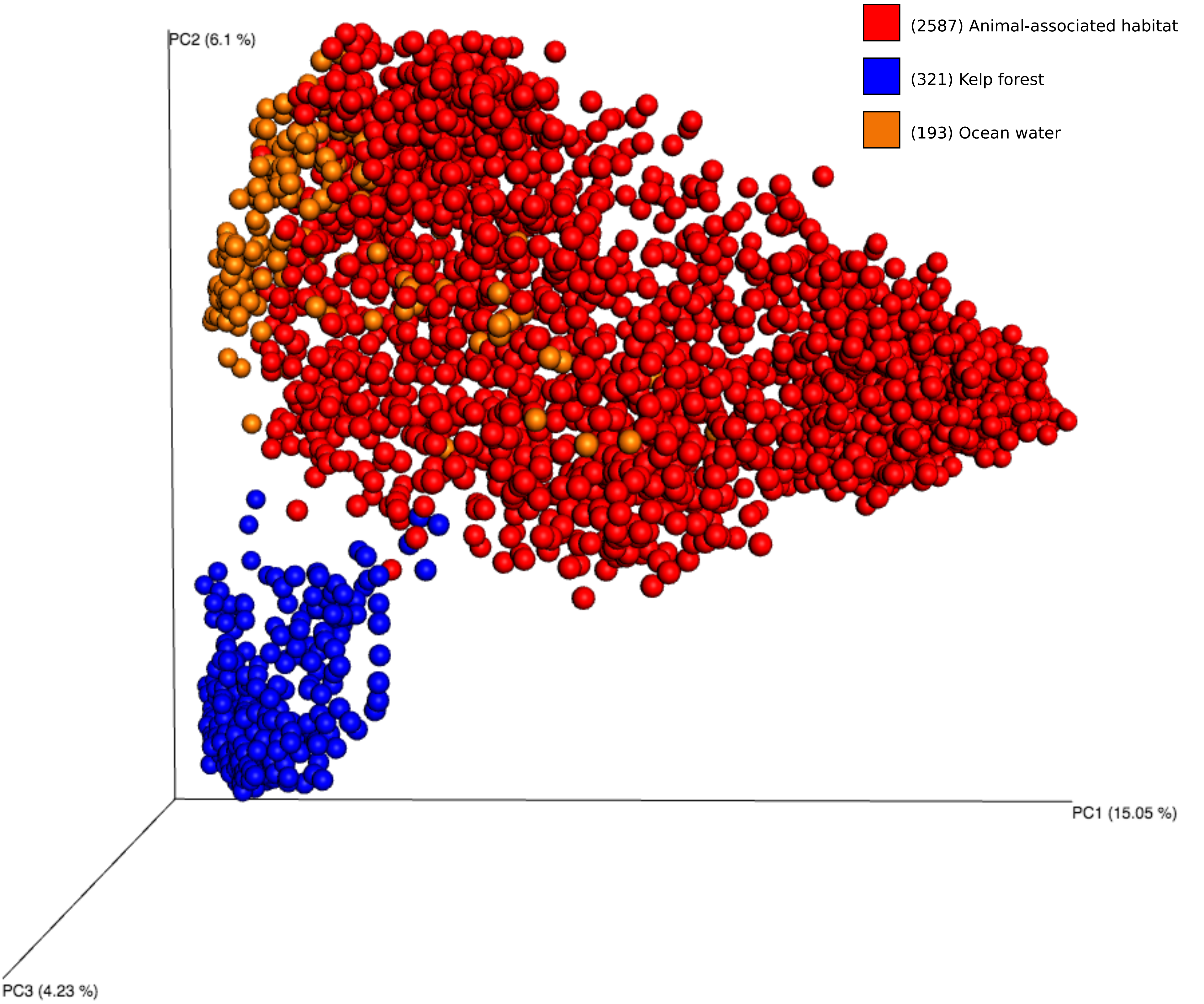

Figure 4

Click here to download Figure Figure.4.png

taxonomy: k__Bacteria;p__Proteobacteria;c__Alphaproteobacteria;o__Rhizobiales

sequence: TACGAAGGGGGCTAGCGTTGTTCGGAATCACTGGGCGTAAAGCGCACGTAGGCGGACTTTTAAGTCAGGGGTGAAATCCCGGGGCTCAACCCCGGAACTG
More info from dbBact
Present in 0.034474 of samples (132 / 3829)

▼ host_scientific_name (6 significant)

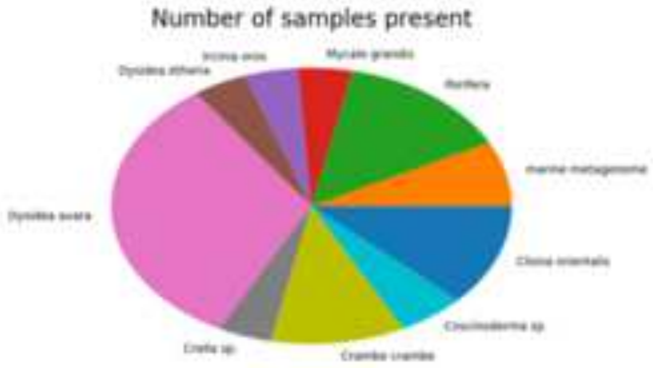

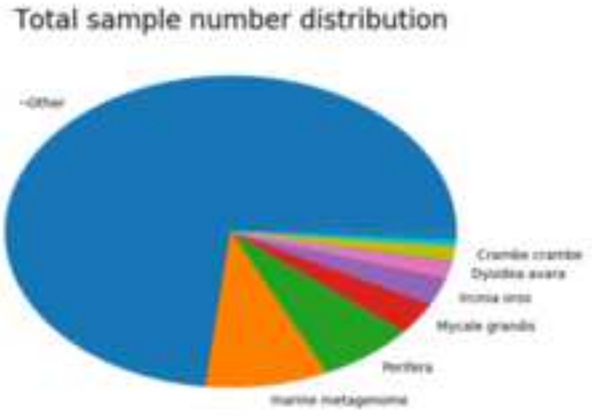

Significant enrichment:
host_scientific_name:Dysidea avara (30/64) (p=0.000000)
host_scientific_name:Crella sp. (4/9) (p=0.000155)
host_scientific_name:Dysidea etheria (4/10) (p=0.000251)
host_scientific_name:Cliona orientalis (11/31) (p=0.000000)
host_scientific_name:Coscinoderma sp. (5/27) (p=0.002082)
host_scientific_name:Crambe crambe (10/56) (p=0.000020)

▶ env_feature (1 significant)
▶ country (3 significant)
▶ ALL (84 significant)
View as table

Movie for Figure 3

Click here to access/download
Supplementary Material
Figure3.movie.gif

Supplementary Table S1

Click here to access/download
Supplementary Material
sample.metadata.tsv

Explanation of Supplementary data that will be uploaded later

Click here to access/download
Supplementary Material
README.txt

Dear Dr. Nogoy,

We thank you for the assessment of the manuscript "The sponge microbiome project" (GIGA-D-17-00079). We have addressed the reviewers' comments as outlined below and hope you find the manuscript now suitable for publication.

Please do not hesitate to contact us with any further questions or comments.

Best wishes,

Torsten Thomas

Reviewer reports:
Reviewer #1: General comments:

Moitinho-Silva et al presented a comprehensive microbiome dataset based on 16S rRNA gene sequencing of 269 sponge host species, along with samples from their habitats of seawater and sediments. With a global sampling coverage and consistent sample handling protocol from sponge tissue collection to DNA extraction, PCR condition and sequencing, this dataset provides a great platform to understand sponge microbiome in spatial and temporal scales. The systematic analysis done here will greatly benefit the sponge microbiome community, also serve as a valuable resource to compare with other host-associated microbiome systems.
In this manuscript, authors described details of the sequencing data analysis pipeline and compared the outcomes from commonly used clustering methods and different reference databases. Accompanied metadata file is well organized and provides valuable information for further meta-analysis.
Although part of the dataset is associated with an analysis article published last year (Thomas, T. et al. 2016), current dataset include more samples and the authors provide additional value by creating the enrichment analysis tool on the website SpongeEMP.

Specific comments:

Line 108: "unique insight" or "insights"

Response:
Only "insights" was kept

Line 120: Were OTUs from negative control samples filtered out from downstream analysis?

Response:
Negative controls were kept in the final dataset to enable user to perform their own analysis of putative contaminating OTUs.

Line 127-133: Some detail information on QIIME pipeline is missing in this section (compare to the information provided in the mothur section below). I tried to find it in the supplementary file but maybe I missed it. How were the sequences quality filtered (like q score, length, etc)? How were the chimeric sequences detected here? What is the minimum reads to be considered as an OTU? There are both phylogenetic- and OTU-based unweighted distance measures, so it should be clarified which was used? If a phylogenetic unweighted distance was used, how the phylogenetic tree for UniFrac was built?

Response:
We have added the following text that clarifies how the QIIME pipeline works and what parameters were used:

"Raw sequences were demultiplexed and quality controlled following the recommendations of [16]. Quality-filtered, demultiplexed fastq files were processed using the default closed-reference pipeline from QIIME v. 1.9.1. Briefly, sequences were matched against GreenGenes reference database (v.

13_8 clustered at 97% similarity). Sequences that failed to align (e.g. chimeras) were discard, which resulted in a final number of 300,140,110 sequences. Taxonomy assignments and the phylogenetic tree information were taken from the centroids of the reference sequence clusters contain in the GreenGenes reference database. This closed-reference analysis allows for cross-dataset comparisons and direct comparison with the tens of thousands of other samples processed in the EMP and available via the Qiita database [17]."

In supplementary materials, authors provided OTU abundance matrix in from Mothur pipeline. For comparison, I feel authors can include in supplement the OTU table generated by QIIME OTU picking in biom format. Additionally, a phylogenetic tree file may be needed for future users to generate UniFrac PCoA plot like Figure 3. Together with the meta-date file, this can greatly facilitate subsequent analysis by sponge community to assess beta-diversity of the microbiome on specific environment factors or host specificity. Line 161: Is the resulting biom file provided as part of the supplemental material here?

Response:
We now provide the QIIME output in biom format and the tree file as supplementary information.

Figure 2. Which cluster method is used here? Mothur or QIIME? The color scheme for Thaumarchaea is different in greengene from the other two database, need to be consistent. Do author have some general comment regarding the pro and cons of using three reference database?

Response:
We now state that Figure 2 is based on the Mothur-based analysis.
The colour code is based on phylum-level assignments and the phylum Thaumarchaeota has been shown in the same colour for the RDP and Silva database. The terminology "Thaumarchaeota" is used as class in the Greengenes taxonomy, which belongs to the phylum "Crenarchaeota". We therefore think it is appropriate to keep the colours different as they represent different taxonomic assignments.

We also now briefly comment on the use of different database as follows "The inclusion of these taxonomies is helpful considering that they have substantial differences as recently discussed [25]. For example, Greengenes and RDP have the taxon Poribacteria, a prominent sponge-enriched phylum [26], which did not exist in the SILVA version used."

Figure 3. I suggest author provide a 3D movie for the PCoA plot as a supplemental material for better visualization of the whole dataset. Alternative, a 2D plot with 3 panels reflecting PC1 vs PC2, PC1 vs PC3 and PC2 vs PC3 also works.

Response:
We now provide a movie of the PCoA plot now in the supplementary information.

Figure 4. The legend states the piechart is based on "relative abundance", but in the figure it is "absolute abundance". Please clarify it.

Response:
There was a mix-up with the labels. We have fixed this to "Total samples present" as well as changed the label to the second pie chart to "Total sample number distribution". We have also modified the figure legend to clarify the meaning of the two pie charts.

My understanding is that authors only consider the presence or absence of a particular OTU in the enrichment analysis. If possible, I would like to see an additional function for enrichment analysis based on the relative abundance of a particular OTU, since relative abundance provides another angle to evaluate the importance of the bacterial OTU in the community. This probably needs to be done on a dataset with normalized sequencing depth (ie, subsampled to 10,000 reads).

Response:
We thank the referee for this useful suggestion. A non-parameteric (Kruskal-Wallis) relative abundance test has been added to the webserver analysis. All category/value pairs significantly enriched in either of the two tests are now listed in the output, as well as the corresponding p-values. Figure 4 and the Database

metadata category enrichment section have been updated to include this additional analysis. All analysis is performed on a subsampled table (to 5000 reads/sample).

Also, can author also show the p value on the website to reflect the degree of enrichment?

Response:
We thank the referees for this useful suggestion. The two-sided binomial p-value for the absence/presence as well as the Kruskal-Wallis p-value for relative abundance have been added to the results page and the summary table.

From a user's point of view, is there a way to export the analysis results (values from the piechart and number of samples with the OTU query) in text format from the website? It will be really helpful and convenient for the community to further evaluate the dataset.

Response:
We thank the referees for this useful suggestion. We have added a link from the results page to an html table summarizing the enrichment results, which can be copied and pasted to excel for further processing.

Reviewer #2: This is a robust dataset for an increasingly important microbiome. The authors present their dataset and describe their data in a clear and concise way.
Some minor (except the last one) issues that need to be clarified are:

1. How was the sponge sampling designed? Was it a random sampling of sponge species found in a certain habitat?

Response:
The sample contributors collected specimen often with specific questions or designs in mind, which will be subject of future publications using the presented dataset.

2. What about the unidentified sponge species? Isn't the unidentified species dataset an impediment in the sponge microbiome comparisons?

Response:
Unidentified species in the context of our study means that the species have not been given a formal taxonomic assignment. This taxonomic assignment is work in progress, which requires quite lengthy procedures, and the outcome of this will be added to the metadata in the future. We decided to still include those samples our study as they can help to address taxa-independent question, such as the occurrence of certain microbes in particular geographic regions.

3. lines 132-133: "Sequences that failed to align were discarded". How many were those sequences id est what is the percentage of sequences used to produce the microbial taxonomic profile of marine sponge samples?

Response:
We provide now the number of sequences (300,140,110) used for the final analysis.

4. lines 209-211: "Raw sequence data were deposited in the European Nucleotide Archive (accession numbers: ERP020690). Quality-filtered, demultiplexed fastq files, Deblur and QIIME resulting OTU tables are available at Qiita database [16] (Study ID: 10793)". No results found for ERP020690 in ENA or Study ID: 10793 in Qiita? Why?

Response:
The data have now been made public.
