# Supplementary figures and images for: The sponge microbiome project
Source: Gigascience. 2017 Aug 16;6(10):gix077. doi: 10.1093/gigascience/gix077 (PMC5632291; doi:10.1093/gigascience/gix077)

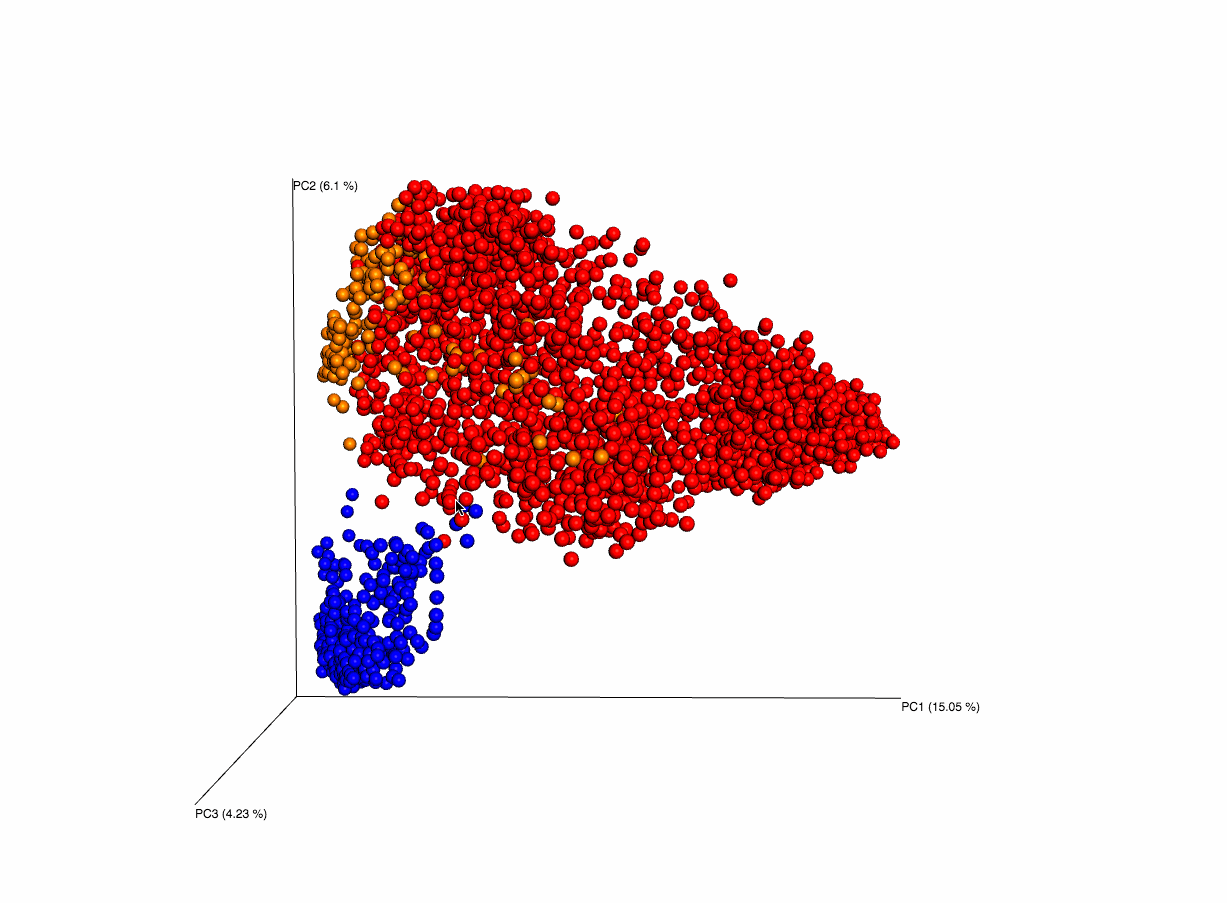

Supplement: Additional Files [file gix077_supp.zip › Figure3.movie.gif]
