# Supplementary material for: The sponge microbiome project
Source: Gigascience. 2017 Aug 16;6(10):gix077. doi: 10.1093/gigascience/gix077 (PMC5632291; doi:10.1093/gigascience/gix077)
Supplement: Response-to-Reviewer-Comments_Original-Submission.pdf [file gix077_response-to-reviewer-comments_original-submission.pdf]

Author's Response To Reviewer Comments

Dear Dr. Nogoy,

We thank you for the assessment of the manuscript "The sponge microbiome project" (GIGA-D-17-00079). We have addressed the reviewers' comments as outlined below and hope you find the manuscript now suitable for publication.

Please do not hesitate to contact us with any further questions or comments.

Best wishes,

Torsten Thomas

Reviewer reports:
Reviewer #1: General comments:

Moitinho-Silva et al presented a comprehensive microbiome dataset based on 16S rRNA gene sequencing of 269 sponge host species, along with samples from their habitats of seawater and sediments. With a global sampling coverage and consistent sample handling protocol from sponge tissue collection to DNA extraction, PCR condition and sequencing, this dataset provides a great platform to understand sponge microbiome in spatial and temporal scales. The systematic analysis done here will greatly benefit the sponge microbiome community, also serve as a valuable resource to compare with other host-associated microbiome systems.
In this manuscript, authors described details of the sequencing data analysis pipeline and compared the outcomes from commonly used clustering methods and different reference databases. Accompanied metadata file is well organized and provides valuable information for further meta-analysis.
Although part of the dataset is associated with an analysis article published last year (Thomas, T. et al. 2016), current dataset include more samples and the authors provide additional value by creating the enrichment analysis tool on the website SpongeEMP.

Specific comments:

Line 108: "unique insight" or "insights"

Response:
Only "insights" was kept

Line 120: Were OTUs from negative control samples filtered out from downstream analysis?

Response:

Negative controls were kept in the final dataset to enable user to perform their own analysis of putative contaminating OTUs.

Line 127-133: Some detail information on QIIME pipeline is missing in this section (compare to the information provided in the mothur section below). I tried to find it in the supplementary file but maybe I missed it. How were the sequences quality filtered (like q score, length, etc)? How were the chimeric sequences detected here? What is the minimum reads to be considered as an OTU? There are both phylogenetic- and OTU-based unweighted distance measures, so it should be clarified which was used? If a phylogenetic unweighted distance was used, how the phylogenetic tree for UniFrac was built?

Response:
We have added the following text that clarifies how the QIIME pipeline works and what parameters were used:

"Raw sequences were demultiplexed and quality controlled following the recommendations of [16]. Quality-filtered, demultiplexed fastq files were processed using the default closed-reference pipeline from QIIME v. 1.9.1. Briefly, sequences were matched against GreenGenes reference database (v. 13_8 clustered at 97% similarity). Sequences that failed to align (e.g. chimeras) were discard, which resulted in a final number of 300,140,110 sequences. Taxonomy assignments and the phylogenetic tree information were taken from the centroids of the reference sequence clusters contain in the GreenGenes reference database. This closed-reference analysis allows for cross-dataset comparisons and direct comparison with the tens of thousands of other samples processed in the EMP and available via the Qiita database [17]."

In supplementary materials, authors provided OTU abundance matrix in from Mothur pipeline. For comparison, I feel authors can include in supplement the OTU table generated by QIIME OTU picking in biom format. Additionally, a phylogenetic tree file may be needed for future users to generate UniFrac PCoA plot like Figure 3. Together with the meta-date file, this can greatly facilitate subsequent analysis by sponge community to assess beta-diversity of the microbiome on specific environment factors or host specificity. Line 161: Is the resulting biom file provided as part of the supplemental material here?

¬¬¬¬¬¬¬¬¬¬
Response:
We now provide the QIIME output in biom format and the tree file as supplementary information.

Figure 2. Which cluster method is used here? Mothur or QIIME? The color scheme for Thaumarchaea is different in greengene from the other two database, need to be consistent. Do author have some general comment regarding the pro and cons of using three reference database?

Response:
We now state that Figure 2 is based on the Mothur-based analysis.
The colour code is based on phylum-level assignments and the phylum Thaumarchaeota has

been shown in the same colour for the RDP and Silva database. The terminology "Thaumarchaeota" is used as class in the Greengenes taxonomy, which belongs to the phylum "Crenarchaeota". We therefore think it is appropriate to keep the colours different as they represent different taxonomic assignments.

We also now briefly comment on the use of different database as follows "The inclusion of these taxonomies is helpful considering that they have substantial differences as recently discussed [25]. For example, Greengenes and RDP have the taxon Poribacteria, a prominent sponge-enriched phylum [26], which did not exist in the SILVA version used."

Figure 3. I suggest author provide a 3D movie for the PCoA plot as a supplemental material for better visualization of the whole dataset. Alternative, a 2D plot with 3 panels reflecting PC1 vs PC2, PC1 vs PC3 and PC2 vs PC3 also works.

Response:
We now provide a movie of the PCoA plot now in the supplementary information.

Figure 4. The legend states the piechart is based on "relative abundance", but in the figure it is "absolute abundance". Please clarify it.

Response:
There was a mix-up with the labels. We have fixed this to "Total samples present" as well as changed the label to the second pie chart to "Total sample number distribution". We have also modified the figure legend to clarify the meaning of the two pie charts.

My understanding is that authors only consider the presence or absence of a particular OTU in the enrichment analysis. If possible, I would like to see an additional function for enrichment analysis based on the relative abundance of a particular OTU, since relative abundance provides another angle to evaluate the importance of the bacterial OTU in the community. This probably needs to be done on a dataset with normalized sequencing depth (ie, subsampled to 10,000 reads).

Response:
We thank the referee for this useful suggestion. A non-parameteric (Kruskal-Wallis) relative abundance test has been added to the webserver analysis. All category/value pairs significantly enriched in either of the two tests are now listed in the output, as well as the corresponding p-values. Figure 4 and the Database metadata category enrichment section have been updated to include this additional analysis. All analysis is performed on a subsampled table (to 5000 reads/sample).

Also, can author also show the p value on the website to reflect the degree of enrichment?

Response:
We thank the referees for this useful suggestion. The two-sided binomial p-value for the absence/presence as well as the Kruskal-Wallis p-value for relative abundance have been added

to the results page and the summary table.

From a user's point of view, is there a way to export the analysis results (values from the piechart and number of samples with the OTU query) in text format from the website? It will be really helpful and convenient for the community to further evaluate the dataset.

Response:
We thank the referees for this useful suggestion. We have added a link from the results page to an html table summarizing the enrichment results, which can be copied and pasted to excel for further processing.

Reviewer #2: This is a robust dataset for an increasingly important microbiome. The authors present their dataset and describe their data in a clear and concise way.
Some minor (except the last one) issues that need to be clarified are:

1. How was the sponge sampling designed? Was it a random sampling of sponge species found in a certain habitat?

Response:
The sample contributors collected specimen often with specific questions or designs in mind, which will be subject of future publications using the presented dataset.

2. What about the unidentified sponge species? Isn't the unidentified species dataset an impediment in the sponge microbiome comparisons?

Response:
Unidentified species in the context of our study means that the species have not been given a formal taxonomic assignment. This taxonomic assignment is work in progress, which requires quite lengthy procedures, and the outcome of this will be added to the metadata in the future. We decided to still include those samples our study as they can help to address taxa-independent question, such as the occurrence of certain microbes in particular geographic regions.

3. lines 132-133: "Sequences that failed to align were discarded". How many were those sequences id est what is the percentage of sequences used to produce the microbial taxonomic profile of marine sponge samples?

Response:
We provide now the number of sequences (300,140,110) used for the final analysis.

4. lines 209-211: "Raw sequence data were deposited in the European Nucleotide Archive (accession numbers: ERP020690). Quality-filtered, demultiplexed fastq files, Deblur and QIIME resulting OTU tables are available at Qiita database [16] (Study ID: 10793)". No results found for ERP020690 in ENA or Study ID: 10793 in Qiita? Why?

Response:
The data have now been made public.
