# Supplementary material for: The sponge microbiome project
Source: Gigascience. 2017 Aug 16;6(10):gix077. doi: 10.1093/gigascience/gix077 (PMC5632291; doi:10.1093/gigascience/gix077)
Supplement: Reviewer-1-Report-(Original-Submission).pdf [file gix077_reviewer-1-report-(original-submission).pdf]

Reviewer Report

Title: The sponge microbiome project

Version: Original Submission **Date:** 4/29/2017

Reviewer name: Fan Zhang

Reviewer Comments to Author:

General comments:Moitinho-Silva et al presented a comprehensive microbiome dataset based on 16S rRNA gene sequencing of 269 sponge host species, along with samples from their habitats of seawater and sediments. With a global sampling coverage and consistent sample handling protocol from sponge tissue collection to DNA extraction, PCR condition and sequencing, this dataset provides a great platform to understand sponge microbiome in spatial and temporal scales. The systematic analysis done here will greatly benefit the sponge microbiome community, also serve as a valuable resource to compare with other host-associated microbiome systems.In this manuscript, authors described details of the sequencing data analysis pipeline and compared the outcomes from commonly used clustering methods and different reference databases. Accompanied metadata file is well organized and provides valuable information for further meta-analysis.Although part of the dataset is associated with an analysis article published last year (Thomas, T. et al. 2016), current dataset include more samples and the authors provide additional value by creating the enrichment analysis tool on the website SpongeEMP.Specific comments:Line 108: "unique insight" or "insights"Line 120: Were OTUs from negative control samples filtered out from downstream analysis?Line 127-133: Some detail information on QIIME pipeline is missing in this section (compare to the information provided in the mothur section below). I tried to find it in the supplementary file but maybe I missed it.How were the sequences quality filtered (like q score, length, etc)? How were the chimeric sequences detected here? What is the minimum reads to be considered as an OTU?There are both phylogenetic- and OTU-based unweighted distance measures, so it should be clarified which was used? If a phylogenetic unweighted distance was used, how the phylogenetic tree for UniFrac was built?In supplementary materials, authors provided OTU abundance matrix in from Mothur pipeline. For comparison, I feel authors can include in supplement the OTU table generated by QIIME OTU picking in biom format. Additionally, a phylogenetic tree file may be needed for future users to generate UniFrac PCoA plot like Figure 3. Together with the meta-date file, this can greatly facilitate subsequent analysis by sponge community to assess beta-diversity of the microbiome on specific environment factors or host specificity.Line 161: Is the resulting biom file provided as part of the supplemental material here?Figure 2. Which cluster method is used here? Mothur or QIIME? The color scheme for Thaumarchaea is different in greengene from the other two database, need to be consistent. Do author have some general comment regarding the pro and cons of using three reference database?Figure 3. I suggest author provide a 3D movie for the PCoA plot as a supplemental material for better visualization of the whole dataset. Alternative, a 2D plot with 3 panels reflecting PC1 vs PC2, PC1 vs PC3 and PC2 vs PC3 also works.Figure 4. The legend states the piechart is based on "relative abundance", but in the figure it is "absolute abundance". Please clarify it.My understanding is that authors only consider the presence or absence of a particular OTU in the

enrichment analysis. If possible, I would like to see an additional function for enrichment analysis based on the relative abundance of a particular OTU, since relative abundance provides another angle to evaluate the importance of the bacterial OTU in the community. This probably needs to be done on a dataset with normalized sequencing depth (ie, subsampled to 10,000 reads).Also, can author also show the p value on the website to reflect the degree of enrichment?From a user's point of view, is there a way to export the analysis results (values from the piechart and number of samples with the OTU query) in text format from the website? It will be really helpful and convenient for the community to further evaluate the dataset.

Level of Interest

Please indicate how interesting you found the manuscript: An article of importance in its field

Quality of Written English

Please indicate the quality of language in the manuscript: Acceptable

Declaration of Competing Interests

Please complete a declaration of competing interests, considering the following questions:

- Have you in the past five years received reimbursements, fees, funding, or salary from an organisation that may in any way gain or lose financially from the publication of this manuscript, either now or in the future?
- Do you hold any stocks or shares in an organisation that may in any way gain or lose financially from the publication of this manuscript, either now or in the future?
- Do you hold or are you currently applying for any patents relating to the content of the manuscript?
- Have you received reimbursements, fees, funding, or salary from an organization that holds or has applied for patents relating to the content of the manuscript?
- Do you have any other financial competing interests?
- Do you have any non-financial competing interests in relation to this paper?

If you can answer no to all of the above, write 'I declare that I have no competing interests' below. If your reply is yes to any, please give details below.

I declare that I have no competing interests

I agree to the open peer review policy of the journal. I understand that my name will be included on my report to the authors and, if the manuscript is accepted for publication, my named report including any attachments I upload will be posted on the website along with the authors' responses. I agree for my report to be made available under an Open Access Creative Commons CC-BY license

(http://creativecommons.org/licenses/by/4.0/). I understand that any comments which I do not wish to be included in my named report can be included as confidential comments to the editors, which will not be published.

I agree to the open peer review policy of the journal
