# Supplementary material for: The sponge microbiome project
Source: Gigascience. 2017 Aug 16;6(10):gix077. doi: 10.1093/gigascience/gix077 (PMC5632291; doi:10.1093/gigascience/gix077)
Supplement: Reviewer-2-Report-(Original-Submission).pdf [file gix077_reviewer-2-report-(original-submission).pdf]

Reviewer Report

Title: The sponge microbiome project

Version: Original Submission **Date:** 5/9/2017

Reviewer name: Spyros Gkelis

Reviewer Comments to Author:

This is a robust dataset for an increasingly important microbiome. The authors present their dataset and describe their data in a clear and concise way.Some minor (except the last one) issues that need to be clarified are:1. How was the sponge sampling designed? Was it a random sampling of sponge species found in a certain habitat?2. What about the unidentified sponge species? Isn't the unidentified species dataset an impediment in the sponge microbiome comparisons?3. lines 132-133: "Sequences that failed to align were discarded". How many were those sequences id est what is the percentage of sequences used to produce the microbial taxonomic profile of marine sponge samples?4. lines 209-211: "Raw sequence data were deposited in the European Nucleotide Archive (accession numbers: ERP020690). Quality-filtered, demultiplexed fastq files, Deblur and QIIME resulting OTU tables are available at Qiita database [16] (Study ID: 10793)". No results found for ERP020690 in ENA or Study ID: 10793 in Qiita? Why?

Level of Interest

Please indicate how interesting you found the manuscript: An article of importance in its field

Quality of Written English

Please indicate the quality of language in the manuscript: Acceptable

Declaration of Competing Interests

Please complete a declaration of competing interests, considering the following questions:

- Have you in the past five years received reimbursements, fees, funding, or salary from an organisation that may in any way gain or lose financially from the publication of this manuscript, either now or in the future?
- Do you hold any stocks or shares in an organisation that may in any way gain or lose financially from the publication of this manuscript, either now or in the future?
- Do you hold or are you currently applying for any patents relating to the content of the manuscript?
- Have you received reimbursements, fees, funding, or salary from an organization that holds or has applied for patents relating to the content of the manuscript?
- Do you have any other financial competing interests?

- Do you have any non-financial competing interests in relation to this paper?

If you can answer no to all of the above, write 'I declare that I have no competing interests' below. If your reply is yes to any, please give details below.

I declare that I have no competing interests

I agree to the open peer review policy of the journal. I understand that my name will be included on my report to the authors and, if the manuscript is accepted for publication, my named report including any attachments I upload will be posted on the website along with the authors' responses. I agree for my report to be made available under an Open Access Creative Commons CC-BY license (http://creativecommons.org/licenses/by/4.0/). I understand that any comments which I do not wish to be included in my named report can be included as confidential comments to the editors, which will not be published.

I agree to the open peer review policy of the journal
